# Hybridized Love Waves in a Guiding Layer Supporting an Array of Plates with Decorative Endings

**DOI:** 10.3390/ma13071632

**Published:** 2020-04-01

**Authors:** Kim Pham, Agnès Maurel, Simon Félix, Sébastien Guenneau

**Affiliations:** 1 IMSIA, ENSTA ParisTech, 828 Bd des Maréchaux, 91732 Palaiseau, France; 2 Institut Langevin, CNRS, ESPCI ParisTech, 1 rue Jussieu, 75005 Paris, France; agnes.maurel@espci.fr; 3 LAUM, CNRS UMR 6613, Le Mans Université, avenue Olivier Messiaen, 72085 Le Mans, France; simon.felix@univ-lemans.fr; 4 Aix Marseille Univ., CNRS, Centrale Marseille, Institut Fresnel, 13013 Marseille, France; sebastien.guenneau@fresnel.fr

**Keywords:** metamaterial, homogenization, elastic metasurface, time domain analysis, elastic energy

## Abstract

This study follows from Maurel et al., Phys. Rev. B 98, 134311 (2018), where we reported on direct numerical observations of out-of-plane shear surface waves propagating along an array of plates atop a guiding layer, as a model for a forest of trees. We derived closed form dispersion relations using the homogenization procedure and investigated the effect of heterogeneities at the top of the plates (the foliage of trees). Here, we extend the study to the derivation of a homogenized model accounting for heterogeneities at both endings of the plates. The derivation is presented in the time domain, which allows for an energetic analysis of the effective problem. The effect of these heterogeneous endings on the properties of the surface waves is inspected for hard heterogeneities. It is shown that top heterogeneities affect the resonances of the plates, hence modifying the cut-off frequencies of a wave mathematically similar to the so-called Spoof Plasmon Polariton (SPP) wave, while the bottom heterogeneities affect the behavior of the layer, hence modifying the dispersion relation of the Love waves. The complete system simply mixes these two ingredients, resulting in hybrid surface waves accurately described by our model.

## 1. Introduction

The problem of waves propagating in an elastic half-space supporting an array of beams or plates is well known in seismology, where the site–city interaction aims at understanding the interaction of seismic waves with a set of buildings. Starting with the seminal work of Housner [1] (see also [2]), the site–city interaction has been intensively studied numerically [3,4,5] and analytically [6,7,8,9,10,11]. In this context, seismic shields, or metabarriers, have been considered using resonators buried in soil [12,13,14,15] or arrays of trees with a gradient in their heights [16,17,18]. More generally, this configuration is the elastic analog of a corrugated interface able to support surface waves, studied in acoustics [19] and in electromagnetism [20,21], where they are known as Spoof Plasmon Polaritons (SPPs). SPPs play a fundament role in the extraordinary transmission of long wavelength electromagnetic waves through metallic gratings [22,23] and have been studied intensively in the past twenty years for their potential applications in subwavelength optics, data storage, light generation, microscopy, and bio-photonics; see, e.g., [24]. Such similarities between surface waves in electromagnetism and elastodynamics fuel research in seismic metamaterials [25], as they lead to simplified models that see behind the tree that hides the forest [26].

To describe classical SPPs, the homogenization of a stratified medium is an easy and efficient tool [27,28]; the analysis is valid in the low frequency regime, namely owing to the existence of a small parameter measuring the ratio of the array spacing to the typical wavelength, and it provides, at the dominant order, the dispersion relation of SPPs. Thanks to the mathematical analogy between the problem in electromagnetism and in elasticity, this approach was applied in [18] accounting for the presence of a guiding soil layer underlain by an elastic half-space. Simple dispersion relations have been obtained from the effective model for the resulting spoof Love waves, so-called because of the characteristics they share with classical Love waves (surface waves supported by the layer on its own) and SPPs. Next, to account for the presence of heterogeneities (a foliage) at the top of the plates (trees), a hybrid model was used where the homogenization was performed locally (near the top of the plates) at the second order.

**Figure 1:**
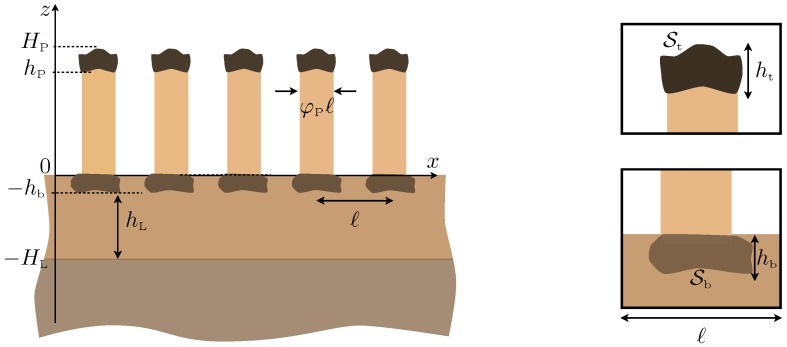
Periodic array of plates decorated at their endings with spacing ℓ=1, height $h_{P}$, and thickness $\varphi_{P}\ell$; the substrate occupying a half-space is surmounted by a guiding layer of thickness $h_{L}$ able to support Love waves. The insets show a zoom on the two endings with heterogeneity surfaces $S_{t}=\varphi_{t}\;h_{t}$ and $S_{b}=\varphi_{b}\;h_{b}$.

**Figure 2:**
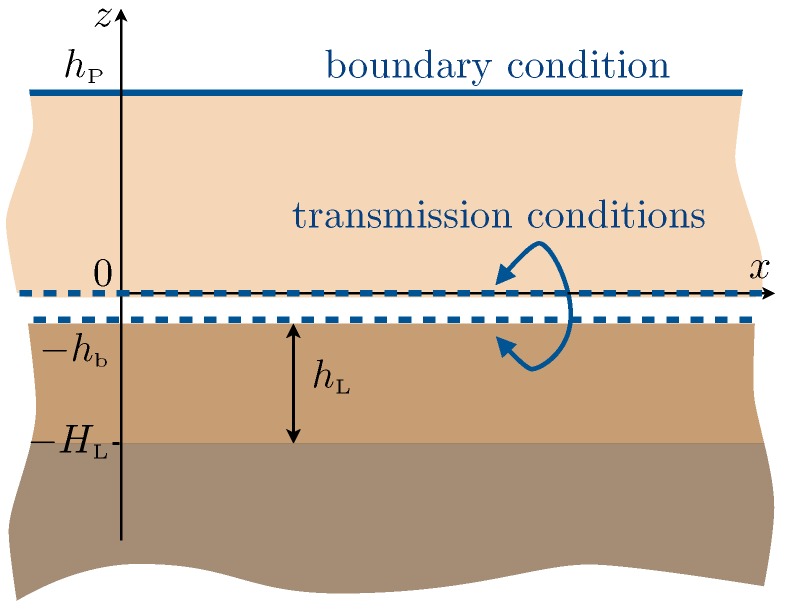
Configuration of the effective problem (2) and (3): The region of the plates has been replaced by a homogeneous medium; the effective boundary condition and transmission conditions encapsulate the effects of the heterogeneities at the decorative endings of the plates.

The present study generalizes and complements this study following two ways: (i) from a physical point of view, we include the effect of heterogeneities at the bottom of the plates (Figure 1), and (ii) from a technical point of view, we derive the full model at second order. This produces a significant increase in the accuracy of the theoretical prediction: in the reported examples, the model at order two is accurate up to a 1–2% error margin, while the model previously used in [18], at order one, would be accurate up to 10–30%. The second order model (see Equations (2) and (3)) provides a one-dimensional problem along the *z*-direction with a succession of homogeneous layers: the substrate occupying a half-space, the guiding layer, and an effective anisotropic layer replacing the region of the plates (see Figure 2). The effect of the heterogeneities at the bottom is encapsulated in transmission conditions, which tell us that the displacement and the normal stress are not continuous; this holds for plates without ending heterogeneities, a fact that was disregarded in [18]. The effect of the heterogeneities at the top is encapsulated in a boundary condition that differs from the usual stress free condition, as in [18]. We recover that for most of the frequencies, the plates do not interact efficiently with the layer; in the present case, it results that the surface wave resembles that of the layer only, hence a wave of the Love type. However, the resonances of the plates produce cut-off frequencies around which the dispersion relations are deeply affected. For simple plates, this can already produce drastic modifications in the dispersion relations (hybridization of the Love branches, avoided crossings at the cut-off frequencies of the plates). When heterogeneities at the endings of the plates are accounted for, additional changes happen. The heterogeneities at the bottom of the plates modify the behavior of the layer on its own, resulting in modified Love waves. The heterogeneities at the top of the plates modify the resonances of the plates, hence the cut-off frequencies. These two simple ingredients allow us to interpret qualitatively the various dispersion relations obtained in the configuration of the plates decorated at both ends. Next, the dispersion relations are accurately recovered by our homogenized model.

The paper is organized as follow. Section 2 summarizes the main results of the analysis: the effective model, Equations (2) and (3), and the resulting equation of energy conservation, Equation (10). The full derivation of the effective model is detailed in the Appendix A and Appendix B. In Section 3, we inspect the characteristics of waves guided by an array of decorated plates. The dispersion relations of these waves are exhibited numerically and compared to the closed forms provided by the effective model, Equations (21)–(23). The heterogeneities have the form of an additional thin hard layer at the bottom of the plates and a thin hard cap on the top. These simple shapes of heterogeneities allow us to discuss the Love waves modified by the bottom heterogeneity only and the resonances of a plate modified by the top heterogeneity.

Throughout the paper, we use the following notations:

- Material properties: mass density ρ and shear modulus μ, with subscripts “S” for the substrate, “L” for the guiding Layer, “P” for the Plates, and “b,t” for the heterogeneities at the bottom and at the top of the plates.
- Geometrical parameters: the layer has a total height $H_{L}=h_{L}+h_{b}$ with $h_{b}$ occupied by the heterogeneities. The array of plates is periodic with spacing *ℓ*, with plate thickness $\varphi_{P}h_{P}$ and total height $H_{P}=h_{P}+h_{t}$ ($h_{t}$ occupied by the heterogeneities). The heterogeneities at the bottom and top of the plates have surfaces $S_{b}=\varphi_{b}h_{b}$ and $S_{t}=\varphi_{t}h_{t}$.

## 2. Summary of the Main Results

In the actual problem, the Navier equations for shear waves simplify to a wave equation for the antiplane displacement $u=u_{y}\left(x,t\right)$ and the stress vector σ(x,t), of the form [29]:

(1) $$\sigma \left(x,t\right)=\mu \left(x\right)\nabla u\left(x,t\right),\;\rho \left(x\right)\frac{\partial^{2}u}{\partial t^{2}}\left(x,t\right)=div\sigma \left(x,t\right),$$

with x=(x,z) and *t* the time. The mass density ρ(x) and the shear modulus μ(x) are piecewise constant in the different materials, substrate/layer/plate/heterogeneities; see Figure 1. At each boundary between the elastic materials, the continuity of the displacement *u* and of the normal stress σ·n holds (with n the local normal vector). Eventually, at the boundaries separating elastic media and air, the stress-free boundary condition σ·n=0 applies. In this section, we present the effective model deduced from the asymptotic analysis developed in Appendix A.

### 2.1. Effective Model

In the effective homogenized model, the regions of the substrate $z\in (-\infty ,-H_{L})$ and of the guiding layer $z\in (-H_{L},-h_{b})$ are kept as in the actual problem, while the region of the plates $z\in (0,h_{P})$ is replaced by an equivalent homogeneous region of the same height. In this region, the medium is highly anisotropic, with propagation being allowed in the vertical direction *z* only; this calculation follows from [18] and applies almost identically in the acoustic case for arrays of Helmholtz resonators [30]. The boundary condition at the top of the effective medium, $z=h_{P}$, is a condition of the Robin type for the normal stress. The transmission conditions at the bottom of the effective medium apply across the actual region of the heterogeneity, and they involve four parameters depending on the geometry of the heterogeneity and of the plates. Specifically, the homogenized model reads as:

(2) $$\left\{\begin{array}{cc} \mathrm{for}z\in (-\infty ,-H_{L}), & \sigma =\mu_{S}\nabla u,\;\rho_{S}\frac{\partial^{2}u}{\partial t^{2}}=\mathrm{div}\sigma ,\\ \mathrm{for}z\in (-H_{L},-h_{b}), & \sigma =\mu_{L}\nabla u,\;\rho_{L}\frac{\partial^{2}u}{\partial t^{2}}=\mathrm{div}\sigma ,\\ \mathrm{for}z\in (0,h_{P}), & \sigma =\mu_{P}\varphi_{P}\left(\begin{array}{cc} 0 & 0\\ 0 & 1 \end{array}\right)\nabla u,\;\rho_{P}\varphi_{P}\frac{\partial^{2}u}{\partial t^{2}}=\mathrm{div}\sigma , \end{array}\right.$$

along with the continuity of *u* and σ·n at $z=-H_{L}$ and the dynamic effective conditions:

(3) $$\left\{\begin{array}{cc} \mathrm{across}\;\mathrm{the}\;\mathrm{region}\;(-h_{b},0), & ⟦u⟧=\frac{\ell_{b}}{\mu_{L}}\;\bar{\sigma_{z}}+l_{b}\;\frac{\partial \bar{u}}{\partial x},\;⟦\sigma_{z}⟧=l_{b}\;\frac{\partial \bar{\sigma_{z}}}{\partial x}-\mu_{L}L_{b}\;\frac{\partial^{2}\bar{u}}{\partial x^{2}}+h_{b}{\hat{\rho }}_{b}\;\frac{\partial^{2}\bar{u}}{\partial t^{2}},\\ \mathrm{at}\;\mathrm{the}\;\mathrm{top}\;\mathrm{of}\;\mathrm{the}\;\mathrm{plates}\;z=h_{P}, & \sigma_{z}\left(x,h_{P},t\right)=-L_{t}\;\frac{\partial \sigma_{z}}{\partial z}\left(x,h_{P},t\right). \end{array}\right.$$

The transmission conditions involve $⟦u⟧=u\left(x,0,t\right)-u\left(x,-h_{b},t\right)$ and $\bar{u}=\frac{1}{2}\left(u\left(x,0,t\right)+u\left(x,-h_{b},t\right)\right)$, being the jump of *u* across the bottom heterogeneity and its mean value, respectively, and the same for $\sigma_{z}$.

**Figure A3:**
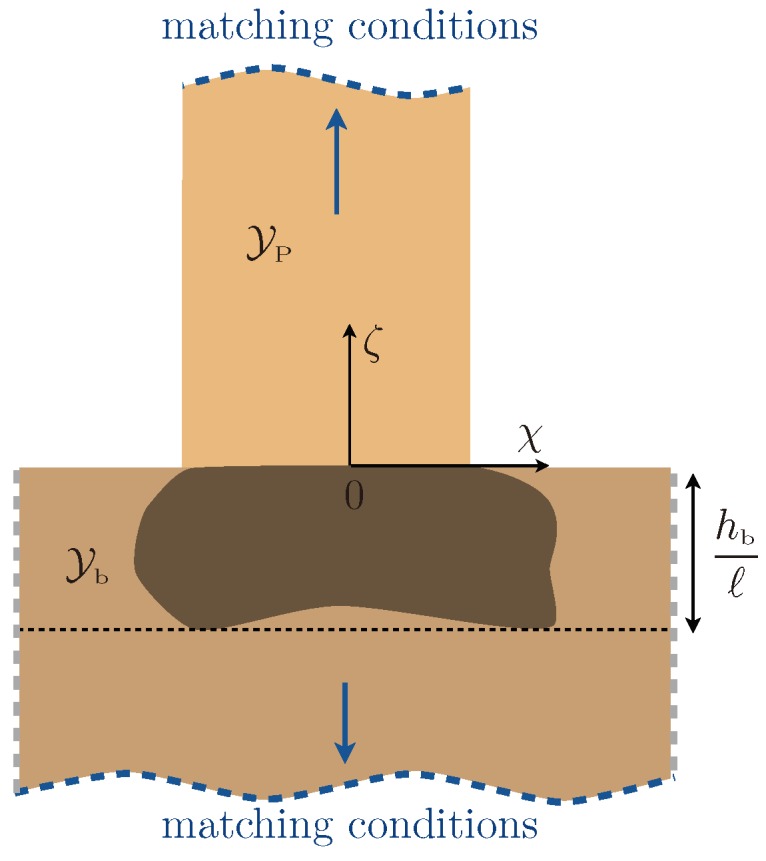
Elementary cell at the bottom of a single plate in the χ=(χ,ζ) coordinate. $Y=Y_{P}\cup Y_{b}$ with $Y_{P}=\left\{\chi \in Y_{P}\times \left(0,+\infty \right)\right\}$, and $Y_{b}=\left\{\chi \in Y\times \left(-\infty ,0\right)\right\}$; $\varphi_{b}h_{b}/\ell$ is the non-dimensional surface of the heterogeneity of vertical extent $h_{b}/\ell$.

Among the five effective parameters $\left(\ell_{b},l_{b},L_{b},{\hat{\rho }}_{b},L_{t}\right)$ entering in the effective conditions, two are known explicitly, while three are defined by elementary problems on $\left(V_{1},V_{2}\right)$ that satisfy static problems set in non-dimensional coordinate χ=(χ,ζ)=(x/ℓ,z/ℓ) in the vicinity of z=0 (see Figure A3 in Appendix A.3). These problems read as:

(4) $$\left\{\begin{array}{ccc} \mathrm{div}\left(\frac{\mu }{\mu_{L}}\nabla V_{1}\right)=0, & \lim_{\zeta \to -\infty }\nabla V_{1}=e_{z}, & \;\lim_{\zeta \to +\infty }\nabla V_{1}=\frac{\mu_{L}}{\varphi_{P}\mu_{P}}e_{z},\\ \mathrm{div}\left(\frac{\mu }{\mu_{L}}\nabla \left(V_{2}+\chi \right)\right)=0, & \lim_{\zeta \to -\infty }\nabla V_{2}=0, & \;\lim_{\zeta \to +\infty }\nabla V_{2}=-e_{x}, \end{array}\right.$$

with $V_{1}$, $\mu \nabla V_{1}\cdot n$ continuous at each interface between two elastic media and $\nabla V_{1}\cdot n=0$ at the boundaries with the air and $V_{1}$ and $\mu \nabla V_{1}$ one periodic with respect to χ for ζ<0 (the same for $V_{2}$ and $\mu \nabla \left(V_{2}+\chi \right)$). Then, we have:

(5) $$\mathrm{Effective}\;\mathrm{parameters}\;\mathrm{in}\;(3)\;\left\{\begin{array}{c} \ell_{b}=\ell \lim_{\zeta \to +\infty }\left(V_{1}-\frac{\mu_{L}}{\varphi_{P}\mu_{P}}\zeta \right)+h_{b},\\ l_{b}=\ell \lim_{\zeta \to +\infty }\left(V_{2}+\chi \right),\\ L_{b}=\ell \int_{Y_{P}}\frac{\mu_{P}}{\mu_{L}}\frac{\partial }{\partial \chi }\left(V_{2}+\chi \right)\;d\chi \;+\ell \int_{Y_{b}}\frac{\mu }{\mu_{L}}\frac{\partial }{\partial \chi }V_{2}\;d\chi +\frac{h_{b}}{\mu_{L}}\left[\varphi_{b}\mu_{b}+\left(1-\varphi_{b}\right)\mu_{L}\right],\\ {\hat{\rho }}_{b}=\varphi_{b}\rho_{b}+\left(1-\varphi_{b}\right)\rho_{L},\\ L_{t}=h_{t}\frac{\rho_{t}\varphi_{t}}{\rho_{P}\varphi_{P}}. \end{array}\right.$$

It is worth noting that the homogenized problem is set in a domain where the regions $\left(-h_{b},0\right)$ and $\left(h_{P},H_{P}\right)$ occupied by the heterogeneities have disappeared. It should be possible to extend the anisotropic region to $\left(0,H_{P}\right)$ as done in [18]; this would lead to a different, but as accurate effective model, with slightly different boundary condition at $z=H_{P}$ (specifically, a different value of $L_{t}$). However, this is not suitable from an energetic point of view (see Section 2.2). Similarly, the transmission conditions involve jumps of the displacement and of the normal stress across a non-zero interface. It should be possible to express the transmission conditions across a zero thickness interface located say at z=0. Again, this would lead to a different and as accurate effective model, with slightly different transmission conditions (with different values of $\ell_{b}$ and $L_{b}$); again, our choice guaranties good properties of the energy in the effective problem.

### 2.2. Effective Energy

The solution (u,σ) of the homogeneous problem is expected to approximate the, say numerical, solution $\left(u_{\mathrm{num}},\sigma_{\mathrm{num}}\right)$ of the actual problem. Hence, we expect that the actual elastic energy is also correctly approximated in the effective problem. In the actual problem, the elastic energy simply reads as [29]:

(6) $$E_{\mathrm{num}}=\frac{1}{2}\int_{D_{\mathrm{num}}}\left(\frac{1}{\mu }{\left|\sigma_{\mathrm{num}}\right|}^{2}+\rho {\left(\frac{\partial u_{\mathrm{num}}}{\partial t}\right)}^{2}\right)\;dx.$$

**Figure 3:**
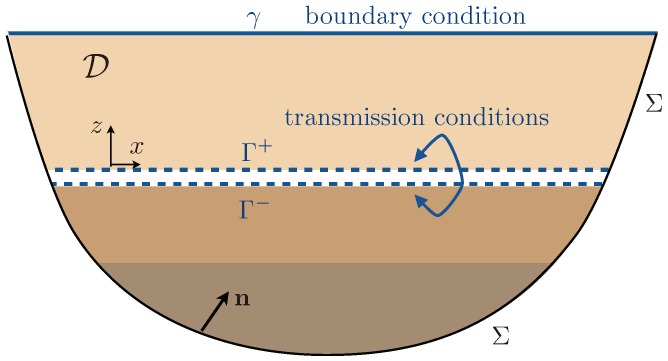
Domain D where the energy is conserved in the absence of incoming/outcoming fluxes through Σ. The effective boundary condition on γ and jump conditions between $\Gamma^{\pm }$ in (3) result in additional effective energies $E_{t,b}$ in (10).

We shall now interrogate the equation of energy conservation in the homogenized problem where the effective boundary and jump conditions in (3) make additional energies appear. These terms appear primarily as fluxes within the bounded region D (see Figure 3), but they can be written as the time derivative of effective energies supported by the surface γ at the top of the plates and across the heterogeneities at the bottom of the plates ($\Gamma^{\pm }$).

By simple manipulation of the equations in (2), the equation of energy conservation in the homogenized problem is found to be of the form:

(7) $$\frac{d}{dt}\left(E_{S}+E_{L}+E_{P}\right)+\Phi =0,$$

with:

(8) $$E_{S,L}=\frac{1}{2}\int_{D_{S,L}}\left(\frac{{\left|\sigma \right|}^{2}}{\mu_{S,L}}+\rho_{S,L}{\left(\frac{\partial u}{\partial t}\right)}^{2}\right)\;dx,\;E_{P}=\frac{1}{2}\int_{D_{P}}\left(\frac{|\sigma_{z}{|}^{2}}{\mu_{P}\varphi_{P}}+\rho_{P}\varphi_{P}{\left(\frac{\partial u}{\partial t}\right)}^{2}\right)\;dx,$$

and:

(9) $$\Phi =\int_{\partial D}\frac{\partial u}{\partial t}\;\sigma \cdot n\;dl$$

(here, Φ is a line integral). The flux Φ has a contribution on Σ and two contributions that do not cancel even if the region D is bounded, that is if Σ is associated with Neumann or Dirichlet boundary conditions. Specifically, $\Phi =\Phi_{\Sigma }+\Phi_{b}+\Phi_{t}$ with:

(10) $$\begin{array}{c} \Phi_{b,t}=\frac{d}{dt}\;E_{b,t}\;\mathrm{and},\\ \;E_{b}=\frac{1}{2}\int_{\Gamma }\;\left[\mu_{L}L_{b}{\left(\frac{\partial \bar{u}}{\partial x}\right)}^{2}+h_{b}{\hat{\rho }}_{b}{\left(\frac{\partial \bar{u}}{\partial t}\right)}^{2}+\frac{\ell_{b}}{\mu_{L}}\;{\bar{\sigma_{z}}}^{2}\right]\;dx,\;E_{t}=\frac{1}{2}\int_{\gamma }\;\rho_{P}\varphi_{P}L_{t}{\left(\frac{\partial u}{\partial t}\right)}^{2}dx, \end{array}$$

where n is the normal interior and $D_{S,L,P}$ the parts of D occupied by the substrate, the layer, and the plates, respectively. We have used that $\sigma_{z}=-L_{t}\rho_{P}\varphi_{P}\partial_{tt}u$ on γ from (2) and (3). We also have that $\Phi_{b}=\int_{\Gamma }\left(\partial_{t}\bar{u}\;⟦\sigma_{z}⟧+\partial_{t}⟦u⟧\;\bar{\sigma_{z}}\right)dx$; hence, $\Phi_{b}\;=\;\int_{\Gamma }\left(\left[l_{b}\partial_{x}\bar{\sigma_{z}}-\mu_{L}L_{b}\partial_{xx}\bar{u}+h_{b}{\hat{\rho }}_{b}\partial_{tt}\bar{u}\right]\partial_{t}\bar{u}+\left[\frac{\ell_{b}}{\mu_{L}}\partial_{t}\bar{\sigma_{z}}+l_{b}\partial_{xt}\bar{u}\right]\bar{\sigma_{z}}\right)dx$. The two terms in $l_{b}$ cancel after integration by parts of one of them, and we integrate also by parts the term in $L_{b}$. It is worth noting that the integrations by parts make boundary terms (b.t.) appear. These terms can be interpreted as concentrated forces at the ending points of $\Gamma^{\pm }$ along *x*; they are disregarded in the present study. Next, $E_{b,t}$ in (10) are energies since they are definite positive quadratic forms. Indeed, $L_{t}>0$ from (5) and ${\hat{\rho }}_{b}>0$ from (5), and it is shown in Appendix B that $\ell_{b}$ and $L_{b}$ are positive as well. It is also worth mentioning that choosing a different position for γ would produce a different and possibly negative value of $L_{t}$. Similarly, expressing the transmission conditions across a zero thickness interface would produce a possibly negative value of $L_{b}$. Discussions on the effective energies can be found in [31,32].

We further stress that the homogenized problem is set on D, which differs from $D_{\mathrm{num}}$; the regions $D_{b}$ for $z\in (-h_{b},0)$ and $D_{t}$ for $z\in (h_{P},H_{P})$ are missing. Intuitively, we expect that the effective energies $E_{b,t}$ represent the elastic energies in $D_{b}$ and $D_{t}$ in the actual problem; specifically, we expect that:

(11) $$E_{b,t}≃\frac{1}{2}\int_{D_{b,t}}\left(\frac{1}{\mu }{\left|\sigma_{\mathrm{num}}\right|}^{2}+\rho {\left(\frac{\partial u_{\mathrm{num}}}{\partial t}\right)}^{2}\right)\;dx.$$

We shall illustrate in Section 3.4 that these intuitive relations are indeed legitimate.

## 3. Hybrid Love Waves in a Guiding Layer Supporting Decorated Plates

**Figure 4:**
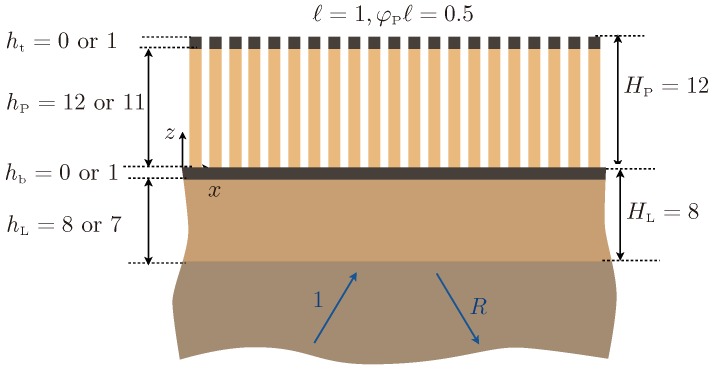
Configuration of the array. The total thickness $H_{P}=h_{P}+h_{b}=12$ of the array and the total thickness $H_{L}=h_{L}+h_{t}=8$ of the layer are kept constant; ℓ=1 and $\varphi_{b}=1$, $\varphi_{t}=\varphi_{P}=0.5$. When the heterogeneities are considered, $h_{b}=h_{t}=1$.

In this section, we inspect the ability of the effective problem (2) and (3) to reproduce the scattering properties of an actual array. We consider the geometry of Figure 4: ℓ=1 in arbitrary unit length, $\varphi_{P}=\varphi_{t}=0.5$ and $\varphi_{b}=1$. The total heights $H_{P}=h_{P}+h_{t}=12$, $H_{L}=h_{L}+h_{b}=8$ are fixed. When the heterogeneities are considered, we set $h_{t}=1$ (hence, $h_{P}=11$) and/or $h_{b}=1$ (hence, $h_{L}=7$). We give in the tables below the material properties and the values of the effective parameters entering in the effective conditions (3).

We consider the time-harmonic regime with a time dependence $e^{-i\omega t}$, which is omitted in the following, and inspect the solution of a scattering problem for a wave coming from z=−∞ with a wavenumber β along *x*, resulting in a reflected wave with a complex reflection coefficient *R*. This scattering problem allows us to cover the case of an incoming propagating wave, with |R|=1 for $\beta \leq \omega /c_{S}$, and the case of guided waves, when |R|=∞ for $\beta >\omega /c_{S}$. The actual problem has to be solved numerically, and this was done using classical multimodal calculations.

In the rest of this section, we shall use for β the component of the wavenumber along *x* and make use of the following quantities:

(12) $$k_{P}=\frac{\omega }{c_{P}},\;\gamma_{L}=\sqrt{\frac{\omega^{2}}{c_{L}^{2}}-\beta^{2}},\;\gamma_{S}=\sqrt{\frac{\omega^{2}}{c_{S}^{2}}-\beta^{2}},$$

($c_{a}=\sqrt{\mu_{a}/\rho_{a}}$ for a = P, L, S).

### 3.1. Two Reference Solutions

To begin with, we establish two families of reference solutions that will be useful to analyze our problem. The first is that of Love waves supported by a guiding layer on the top of a substrate with $c_{L}<c_{S}$, which can be affected by the presence of the bottom heterogeneities. The second family is that of the Spoof Plasmon Polaritons (SPPs) in the plates, which can be affected by the presence of heterogeneities at the bottom of the plates.

#### 3.1.1. Love Waves and Modified Love Waves

**Figure 5:**
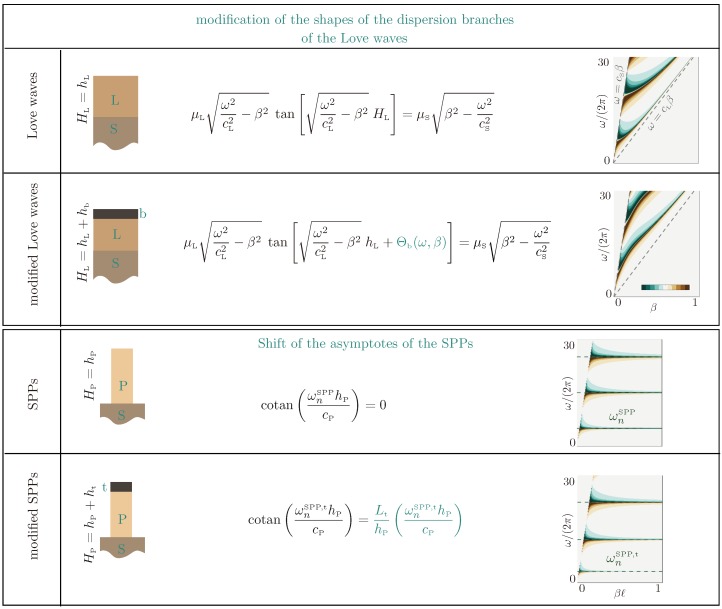
Reference solutions: Dispersion relations of Love waves and modified Love waves (with a bottom layer of thickness $h_{b}$). In the presence of a thin layer b atop the guiding layer, the dispersion relation is modified ($\Theta_{b}$ in (16)) resulting in different shapes of the Love dispersion branches. SPP, Spoof Plasmon Polariton.

If we remove the array (Figure 5), the problem is reduced to a guiding layer sandwiched between air and the semi-infinite substrate (classical Love wave), and its modified version when a thin hard layer is added. The exact solutions of these problems are easily obtained. For classical Love waves, the solution of the scattering problem reads as:

(13) $$u\left(x,z\right)=e^{i\beta x}\times \;\left\{\begin{array}{cc} A\cos\left(\gamma_{L}z\right), & z\in (-h_{L},0),\\ e^{i\gamma_{S}\left(z+h_{L}\right)}+R_{\mathrm{Love}}e^{-i\gamma_{S}\left(z+h_{L}\right)}, & z\in (-\infty ,-h_{L}), \end{array}\right.$$

and using the continuity of the displacement and of the normal stress provides $\left(A,R_{\mathrm{Love}}\right)$, in particular:

(14) $$R_{\mathrm{Love}}=-\frac{\tan\left(\gamma_{L}h_{L}\right)-iY}{\tan\left(\gamma_{L}h_{L}\right)+iY},\;\;\mathrm{with}\;Y=\frac{\mu_{S}\gamma_{S}}{\mu_{L}\gamma_{L}}.$$

We recover the usual dispersion relation of Love waves for *Y* imaginary ($\gamma_{S}$ imaginary with a positive imaginary part) and $|R_{\mathrm{Love}}|=\infty$, which guaranties a family of Love wave dispersion branches in $\omega /c_{S}<\beta <\omega /c_{L}$; see Figure 5.

If we add a layer of thickness $h_{b}$ in the guiding layer, the exact solution reads as:

(15) $$u\left(x,z\right)=e^{i\beta x}\times \;\left\{\begin{array}{cc} A\cos\left(\gamma_{b}z\right), & z\in (-h_{b},0),\\ B\cos\left(\gamma_{L}z\right)+C\sin\left(\gamma_{L}z\right), & z\in (-h_{L},-h_{b}),\\ e^{i\gamma_{S}\left(z+h_{L}\right)}+R_{\mathrm{Love}}^{b}e^{-i\gamma_{S}\left(z+h_{L}\right)}, & z\in (-\infty ,-h_{L}). \end{array}\right.$$

Again, applying the continuity of the displacement and of the normal stress at $z=-h_{L}$, $-h_{b}$ provides $\left(A,B,C,R_{\mathrm{Love}}^{b}\right)$ and, in particular:

(16) $$R_{\mathrm{Love}}^{b}=-\frac{\tan\left(\gamma_{L}h_{L}+\Theta_{b}\right)-iY}{\tan\left(\gamma_{L}h_{L}+\Theta_{b}\right)+iY},\;\Theta_{b}=\tan^{-1}\left(\frac{\mu_{b}\gamma_{b}}{\mu_{L}\gamma_{L}}\tan\left(\gamma_{b}h_{b}\right)\right),$$

where we have defined $\gamma_{b}=\sqrt{\frac{\omega^{2}}{c_{b}^{2}}-\beta^{2}}$, $c_{b}=\sqrt{\mu_{b}/\rho_{b}}$. Surface waves in this configuration have a dispersion relation $\tan\left(\gamma_{L}h_{L}+\Theta_{b}\right)+iY=0$, which can differ significantly from the dispersion relation of the classical Love waves; see Figure 5.

#### 3.1.2. SPPs and Modified SPPs

The dispersion relation of spoof plasmons was derived using approximate methods [19,21] including classical homogenization [27,28]. However, the asymptotes at the cutoff frequencies can be straightforwardly calculated since they correspond to resonances of the plates associated with Dirichlet–Neumann boundary conditions at the bottom-top of the plate. For the classical SPPs, with $k_{P}=\omega /c_{P}$, the solution simply reads as $u\left(x,z\right)=A\cos\left(k_{P}\left(z-H_{P}\right)\right)e^{i\beta x}$ for $z\in (0,H_{P})$, where we have anticipated the Neumann boundary condition (stress-free condition) at the top of the plate. At resonance, the Dirichlet boundary condition applies at z=0 (the plate is clamped to the layer or to the substrate), resulting in the resonance frequency defining the asymptotes for:

(17) $$\mathrm{Asymptotes}\;\mathrm{of}\;\mathrm{SPPs}:\;\;\cot\mathrm{an}\left(k_{P}H_{P}\right)=0,\;\mathrm{hence}\;\omega_{n}^{\mathrm{SPP}}=\left(2n+1\right)\frac{\pi }{2}\frac{c_{P}}{H_{P}}.$$

**Figure 6:**
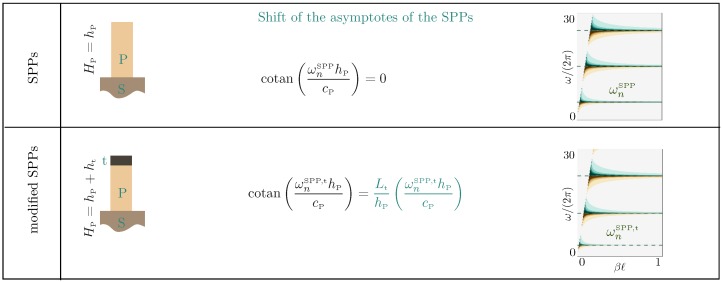
Asymptotes of the SPPs (at $\omega_{n}^{\mathrm{SPP}}/\left(2\pi \right)=3,15,25$) and modified SPPs (at $\omega_{n}^{\mathrm{SPP},t}/\left(2\pi \right)=3.1,12.0,22.4$).

When the plate is terminated by a cap of the same thickness $\varphi_{P}$ and height $h_{t}$ (with $k_{t}\;=\;\omega /c_{t}$), the solution reads as $u\left(x,z\right)\;=\;\left(B\cos\left(k_{P}z\right)+C\sin\left(k_{P}z\right)\right)e^{i\beta x}$ for $z\in (0,h_{P})$ and $u\left(x,z\right)\;=\;A\cos\left(k_{t}\left(z-H_{P}\right)\right)e^{i\beta x}$ for $z\in (h_{P},H_{P})$. Still at resonance, the Dirichlet boundary condition at z=0 imposes B=0; then applying the continuity of the displacement and normal stress at $z=h_{P}$ provides two relations on (A,C), which are compatible if:

(18) $$\mathrm{Asymptotes}\;\mathrm{of}\;\mathrm{modified}\;\mathrm{SPPs}:\;\;\cot\mathrm{an}\left(k_{P}h_{P}\right)=\frac{\mu_{t}k_{t}}{\mu_{P}k_{P}}\tan\left(k_{t}h_{t}\right)≃k_{P}L_{t},$$

and the last equality holds in the case where $k_{t}h_{t}\ll 1$ with $L_{t}$ defined in (5) (and $L_{t}=0$ for $h_{t}=0$). In our geometry, with $L_{t}=10$, $H_{P}=12$, and $c_{P}=240$, the first three asymptotes of the classical SPPs are obtained for $\omega_{n}^{\mathrm{SPP}}/\left(2\pi \right)=3,15,25$. In the presence of the caps of thickness $h_{t}=1$ (hence, $h_{P}=11$), solving the implicit relation $\cot\mathrm{an}X=\frac{10}{11}X$, with $X=\omega h_{P}/c_{P}$, provides the first three modified asymptotes at $\omega_{n}^{\mathrm{SPP},t}/\left(2\pi \right)=3.1,12.0,22.4$; see Figure 6.

### 3.2. Dispersion Relation of Hybridized Love Waves

We shall see that the properties of the hybridized Love waves can be understood in light of the two ingredients studied in the previous section. On the one hand, for weak coupling between the plates and the guiding layer, our surface waves resemble Love waves, which are affected by the presence of heterogeneities. On the other hand, their dispersion relation is deeply modified in the vicinity of the SPPs asymptotes at cut-off frequencies (the coupling is maximum at those frequencies); the cut-off frequencies are dictated by the characteristics of the plates, hence sensitive to the presence of heterogeneities at their tops.

For the geometry of Figure 4, the solution of the scattering problem reads as:

(19) $$u\left(x,z\right)=e^{i\beta x}\times \;\left\{\begin{array}{cc} A\left[\cos\left(k_{P}\left(z-h_{P}\right)\right)+k_{P}L_{t}\sin\left(k_{P}\left(z-h_{P}\right)\right)\right], & z\in (0,h_{P}),\\ B\cos\left(\gamma_{L}\left(z+h_{b}\right)\right)+C\sin\left(\gamma_{L}\left(z+h_{b}\right)\right), & z\in (-H_{L},-h_{b}),\\ e^{i\gamma_{S}\left(z+H_{L}\right)}+Re^{-i\gamma_{S}\left(z+H_{L}\right)}, & z\in (-\infty ,-H_{L}). \end{array}\right.$$

We have accounted for the boundary condition $\sigma_{z}=-L_{t}\partial_{z}\sigma_{z}$ at $z=h_{P}$ (from (3)). Next, accounting for the continuities of the displacement and of the normal stress at $x=-H_{L}$ and for the effective transmission conditions in (3) between $x=-h_{b}$ and x=0 provides the four relations needed to deduce (A,B,C,R). This leaves us with:

(20) $$R=-\frac{D^{*}\left(\omega ,\beta \right)}{D(\omega ,\beta )},$$

where:

(21) $$\begin{array}{cc} D(\omega ,\beta )= & \left[\frac{\varphi_{P}\mu_{P}k_{P}}{\mu_{L}\gamma_{L}}\left(\tan\left(k_{P}h_{P}\right)+k_{P}L_{t}\right)+C_{b}\left(1-k_{P}L_{t}\;\tan\left(k_{P}h_{P}\right)\right)\right]\left(1-iY\tan\left(\gamma_{L}h_{L}\right)\right)\\ & +\left[1-k_{P}L_{t}\tan\left(k_{P}h_{P}\right)-\frac{\varphi_{P}\mu_{P}}{\mu_{L}}k_{P}\ell_{b}\left(\tan\left(k_{P}h_{P}\right)+k_{P}L_{t}\right)\;\right]\left(\tan\left(\gamma_{L}h_{L}\right)+iY\right), \end{array}$$

and:

(22) $$Y=\frac{\mu_{S}\gamma_{S}}{\mu_{L}\gamma_{L}},\;C_{b}=\frac{h_{b}{\hat{\rho }}_{b}\omega^{2}-\mu_{L}L_{b}\beta^{2}}{\mu_{L}\gamma_{L}}.$$

In (20), $D^{*}$ is deduced from *D* by substituting iY by −iY. It results that for waves propagating in the substrate ($\gamma_{L}$ and $\gamma_{S}$ real for $\beta <\omega /c_{S}<\omega /c_{L}$), $D^{*}$ is the complex conjugate of *D*, and |R|=1, as expected. Next, surface waves correspond to $\gamma_{S}$ imaginary with a positive imaginary part, and |R|=∞; hence, we deduce that:

(23) $$\mathrm{Dispersion}\;\mathrm{relation}\;\mathrm{of}\;\mathrm{guided}\;\mathrm{waves}:\;\;D\left(\omega ,\beta \right)=0,\;-i\gamma_{S}\;\mathrm{real}\;>0.$$

It is worth noting that we recover the exact dispersion relations of the classical and modified Love waves for $H_{P}=0$, whence $h_{P}=h_{t}=0$ and $L_{t}=0$ in (5). We still have to determine $C_{b}$ in (22), and to do so, we have to adapt the elementary problem for $V_{2}$ to find $L_{b}$ in (5). This can be done easily by setting $\langle \sigma_{z}^{0}\rangle \left(x,0,t\right)=0$ in (A30) and replacing the limit to +∞ by a boundary condition on χ=0; it results that the limit at +∞ of $V_{2}$ in (4) is replaced by $\nabla V_{2}=-e_{x}$ at ζ=0, and the integral over $Y_{P}$ cancels in (5); the problem is simpler, but still non-trivial. However, in the case where the heterogeneity in the bottom is a thin layer ($\varphi_{b}=1$), the integral over $Y_{b}$ cancels as well by periodicity, and $L_{b}=h_{b}\mu_{b}/\mu_{L}$. Thus, we get $D\left(\omega ,\beta \right)=C_{b}\left(1-iY\left(\tan\gamma_{L}h_{L}\right)\right)+\left(\tan\left(\gamma_{L}h_{L}\right)+iY\right)$, with $C_{b}=\frac{\mu_{b}\gamma_{b}^{2}h_{b}}{\mu_{L}\gamma_{L}}≃\tan\Theta_{b}$. Expectedly, we recover the exact dispersion relation of Love waves for $h_{b}=0$ ($C_{b}=0$) and that of the modified Love waves in the limit of small $h_{b}$.

### 3.3. Validation of the Homogenized Solution

**Figure 7:**
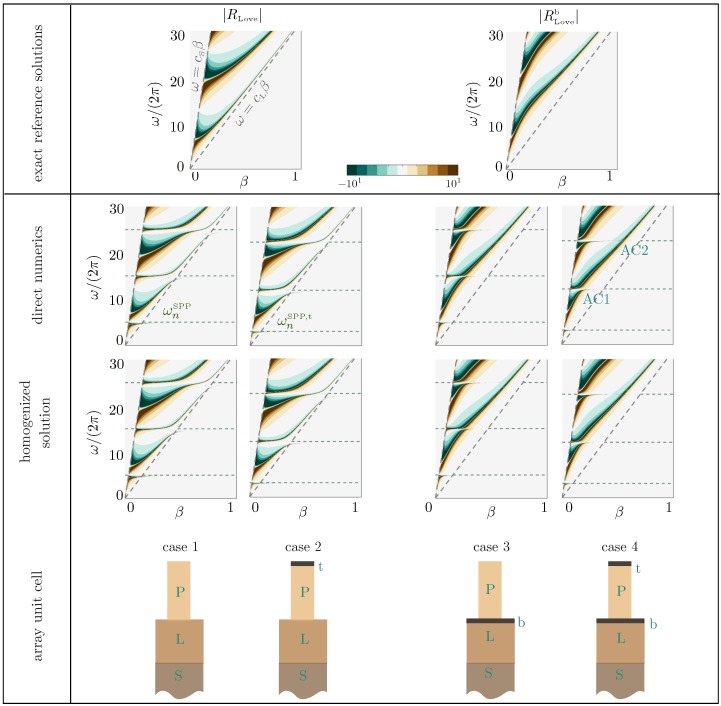
Dispersion relations of guided waves in four configurations of plates from direct numerics and from the homogenized solution (20)–(22); the dispersion relations are revealed by large |R| values. The exact reference dispersion relations of classical and modified Love waves are given in the top panel for comparison. Dotted lines are a guide for the eye showing light lines for Love waves and asymptotes for SPPs. AC1 and AC2 are avoided crossings magnified in Figure 8. Details of homogenized coefficients and geometrical parameters are given in Table 2 and Table 3.

Figure 7 shows the main results of the present study; we report the dispersion relations in four cases from the undecorated plates to the plates decorated at both endings; see the Table 3. They are visible by means of a large (diverging) reflection coefficient in $R_{\mathrm{num}}$ computed in the direct numerics and from the explicit homogenized *R* value in (20)–(22).

The exact dispersion relations of the classical, (14), and modified, (16), Love waves are given for comparison. As previously said, the interaction of the plates with the layer is weak except in the vicinity of the cut-off frequencies. However, for the relatively tall plates that we have considered, these cut-off frequencies are sufficiently close to each other to modify the dispersion relation of our guided waves deeply.

This is already visible for the undecorated plates (Case 1); the guided waves tend to resemble the classical Love waves, but they experience several hybridizations at the cut-off frequencies of the SPPs in (17), accompanied by avoided crossings (sometimes not so pronounced). In the presence of the caps on the top of the plates, the same scenario is observed, with now cut-off frequencies given by the modified SPPs in (18). Eventually, Cases 3 and 4 with the thin hard bottom layer reproduce the same sequence as Cases 1 and 2 with the guided waves, which tend to resemble the modified Love waves.

**Figure 8:**
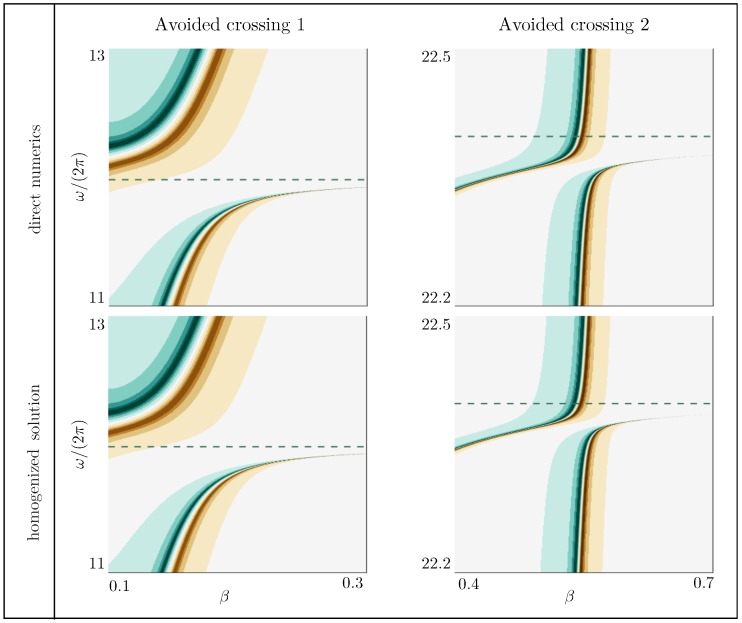
Magnified views of the two avoided crossings AC1 and AC2 from Figure 7 (Case 4).

The ability of our homogenized solution to reproduce the actual dispersion relations accurately is excellent, less than 2% on average in the reported ranges of ω and β, once |R|-values larger than 10 have been saturated. This is particularly visible in the zooms of Figure 8 in the vicinity of avoided crossings for the decorated plates (AC1 and AC2 in Figure 7). In particular, we stress that in the absence of heterogeneity, we have a stress-free condition since $L_{t}=0$ in (5), but we do not have the continuity of the displacement and normal stress across z=0 since $\ell_{b}$ and $L_{b}$ do not vanish (see Table 1). In comparison, the homogenized model (see Table 2 for homogenized coefficients) at the leading order provides the usual stress-free condition and continuity relations regardless of the presence of heterogeneities. Thus, it misses the effects of the heterogeneities and provides the same prediction for Cases 1 to 4. The resulting error is significantly higher, about 10% for Cases 1 and 2, and about 30% for Cases 3 and 4.

**Figure 9:**
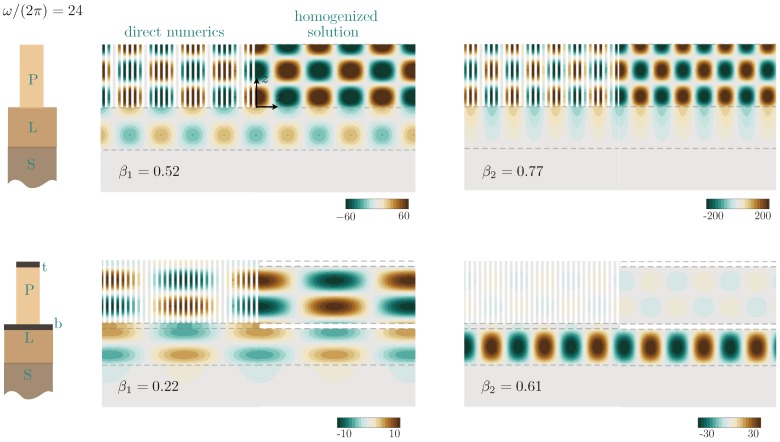
Displacement fields corresponding to the two branches of guided waves ($\beta_{1}$ and $\beta_{2}$) at ω/(2π)=24 for the undecorated plates (top) and decorated plates (bottom). On each panel, the field from direct numerics is plotted for x<0, and the homogenized solution from (19) is plotted for x>0. In both cases, the displacement at $z=-H_{L}$ is unitary, which allows for a quantitative comparison without any tuning parameter.

Eventually, we report in Figure 9 examples of the displacement fields of the guided wave at ω/(2π)=24 for the arrays of undecorated plates and of decorated plates. Both in the numerics and in the homogenized problem (19), the whole solutions were divided by $R_{\mathrm{num}}$ and *R* respectively in order to produce an evanescent wave in the substrate of the form $e^{i\beta x}e^{|\gamma_{S}|\left(x+H_{L}\right)}$ that has the unitary amplitude at $z=-H_{L}$. This allows for quantitative comparison between the direct numerics and the homogenized solution without any adjustable parameter, see Table 2. The agreement in the strengths of the resonances and in the repartition of the amplitudes in the substrate, in the layer, and in the plates is again excellent. At the reported frequency, the wave is evanescent in the substrate as soon as β>0.15, and it becomes evanescent in the layer for β>0.75; this is visible for the undecorated plates for the guided waves with $\beta_{2}=0.77$, which is supported by the array only.

### 3.4. Energies in the Actual/Homogenized Problems

In this section, we inspect the intuitive relations announced in (11), namely that the effective energies $E_{b,t}$ coincide with the elastic energies stored in the regions of the heterogeneities.

In the actual problem, we define $D_{\mathrm{num}}=\left\{x\in \left(0,1\right),z\in \left(-H^{*},0\right)\right\}\cup \left\{x\in \left(0,\varphi_{P}\right),z\in \left(0,H_{P}\right)\right\}$. The energies in the actual problem are the usual elastic energies, which read, in the succession of regions, substrate, layer, bottom, plate, and top, as:

(24) $$\begin{array}{c} E_{\mathrm{num},A}=\frac{1}{2}\;\int_{D_{A}}\;\left(\mu_{\mathrm{num},A}|\nabla u_{\mathrm{num}}{|}^{2}+\rho_{\mathrm{num},A}\omega^{2}{\left|u_{\mathrm{num}}\right|}^{2}\right)\;dx, \end{array}$$

where A = S, L, b, P, t and $D_{S}=\left\{x\in \left(0,1\right),z\in \left(-H^{*},-h_{L}\right)\right\}$, $D_{L}=\left\{x\in \left(0,1\right),z\in \left(-h_{L},-h_{b}\right)\right\}$, $D_{b}=\left\{x\in \left(0,1\right),z\in \left(-h_{b},0\right)\right\}$, $D_{P}=\left\{x\in \left(0,\varphi_{P}\right),z\in \left(0,h_{P}\right)\right\}$, $D_{t}=\left\{x\in \left(0,\varphi_{P}\right),z\in \left(h_{P},H_{P}\right)\right\}$.

In the effective problem, the energies are obtained explicitly owing to the solution in (19), which provides the fields in the substrate, layer, and effective region accounting for the plates. We denote $u\left(x,z\right)=f_{S,P,L}\left(z\right)e^{i\beta x}$ in (19); hence, $f_{S}\left(z\right)=e^{i\gamma_{S}\left(z+H_{L}\right)}+Re^{-i\gamma_{S}\left(z+H_{L}\right)}$, $f_{L}\;=\;B\cos\left(\gamma_{L}\left(z+h_{b}\right)\right)+C\sin\left(\gamma_{L}\left(z+h_{b}\right)\right)$, and $f_{P}\left(z\right)=A\left[\cos\left(k_{P}\left(z-h_{P}\right)\right)+k_{P}L_{t}\sin\left(k_{P}\left(z-h_{P}\right)\right)\right]$, *R* given by (20)–(22), and:

(25) $$\left\{\begin{array}{c} A=\frac{2iY}{D\left(\omega ,\beta \right)\cos\left(k_{P}h_{P}\right)\;\cos\left(\gamma_{L}h_{L}\right)},\\ B=\frac{2iY}{D\left(\omega ,\beta \right)\cos\left(\gamma_{L}h_{L}\right)}\left[1-k_{P}L_{t}\tan k_{P}h_{P}-\frac{\varphi_{P}\mu_{P}}{\mu_{L}}k_{P}\ell_{b}\left(\tan\left(k_{P}h_{P}\right)+k_{P}L_{t}\right)\right],\\ C=\frac{2iY}{D\left(\omega ,\beta \right)\cos\left(\gamma_{L}h_{L}\right)}\left[\frac{\varphi_{P}\mu_{P}k_{P}}{\mu_{L}\gamma_{L}}\left(\tan\left(k_{P}h_{P}\right)+k_{P}L_{t}\right)+C_{b}\left(1-k_{P}L_{t}\;\tan\left(k_{P}h_{P}\right)\right)\right]. \end{array}\right.$$

It follows that the effective energies in (8) read as:

(26) $$\begin{array}{c} E_{S}=\int_{-H^{*}}^{-h_{L}}\left(\mu_{S}|f_{S}^{\prime }{|}^{2}+\left(\rho_{S}\omega^{2}+\mu_{S}\beta^{2}\right){\left|f_{S}\right|}^{2}\right)dz,\;E_{L}=\int_{-h_{L}}^{-h_{b}}\left(\mu_{L}|f_{L}^{\prime }{|}^{2}+\left(\rho_{L}\omega^{2}+\mu_{L}\beta^{2}\right){\left|f_{L}\right|}^{2}\right)dz,\\ E_{P}=\int_{0}^{h_{P}}\left(\mu_{P}\varphi_{P}|f_{P}^{\prime }{|}^{2}+\rho_{P}\varphi_{P}\omega^{2}{\left|f_{P}\right|}^{2}\right)dz, \end{array}$$

and for the last integral, we accounted for the effective stress–displacement relations in (2). Next, from (10) along with (3), it is easy to see that:

(27) $$E_{b}=\frac{\ell }{2}\left[\mu_{L}L_{b}\beta^{2}|f_{P}{\left(0\right)|}^{2}+h_{b}{\hat{\rho }}_{b}\omega^{2}|f_{P}{\left(0\right)|}^{2}+\frac{\ell_{b}}{\mu_{L}}\;{\left(\mu_{P}\varphi_{P}k_{P}\right)}^{2}{\left|f_{P}^{\prime }\left(0\right)\right|}^{2}\right],\;E_{t}=\frac{\ell }{2}\rho_{P}\varphi_{P}L_{t}\omega^{2}{\left|f_{P}\left(h_{P}\right)\right|}^{2}.$$

**Figure 10:**
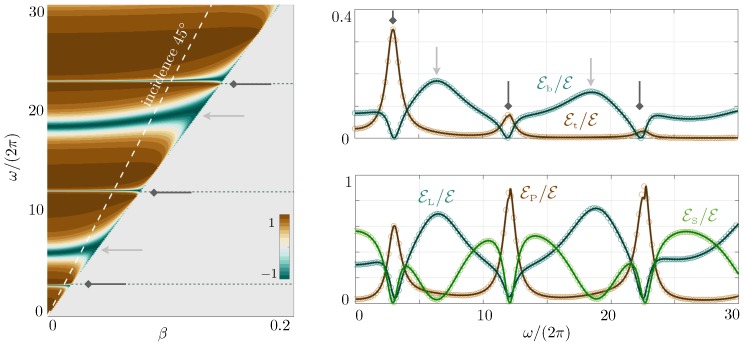
Left: Real part of the reflexion coefficient R∈(−1,1) in colorscale against β and $\omega \;\geq \;c_{S}\beta$. The dashed white line corresponds to an incident propagating wave at oblique incidence with $\beta \;=\;\frac{\omega }{c_{S}}\sin45^{\circ }$. Right: Repartition of the energies in the bottom and top heterogeneities (upper panel) and in the substrate, layer, and plate (normalized with the total energy); see the lower panel. Open symbols are obtained from direct numerics, Equations (24), and plain lines from the homogenized problem, Equations (26) and (27).

We computed the energies in the actual problem, (24), and in the homogenized problem, (26) and (27), for an incident propagating wave ($\gamma_{S}$ real in (19)). The real part of the reflection coefficient *R* is reported in the left panel of Figure 10. In the case of weak coupling with the array of plates, $R≃R_{\mathrm{Love}}^{b}$, (16); hence, R≃1, except in the vicinity of the resonances of the layer (diverging $\tan\left(\gamma_{L}h_{L}+\Theta_{b}\right)$) where it goes to −1; see light grey arrows. Next, strong coupling with the array occurs at the resonance of the plates, resulting in R≃−1; see the dark grey arrows. The resulting repartition of the energies is plotted against the frequency for a wave at incidence $45^{\circ }$ (we normalized the energies to the total energy). For ω/(2π)∈(030), three resonances of the plates and two resonances of the layer take place. Expectedly, the energy in the plates $E_{P}$ is small except at the resonance of the plates where almost all the energy is shared in the plates and their top heterogeneities. This is particularly visible at the first resonance where 35% of the total energy is supported by the heterogeneities. Symmetrically, at the resonances of the layer, most of the energy is supported by the layer ($E_{L}$) and the bottom heterogeneities ($E_{t}$). The ability of the homogenized solution to reproduce the solution in the substrate, in the layer, and in the plates is recovered in the energies with error margins of 0.1%, 0.5%, and 4%, respectively, in the reported case. More remarkably, the effective energies $E_{t}$ and $E_{b}$ accurately reproduce the variations of the actual elastic energies, with error margins of 0.7%, which legitimizes the intuitive relations (11).

**Figure 11:**
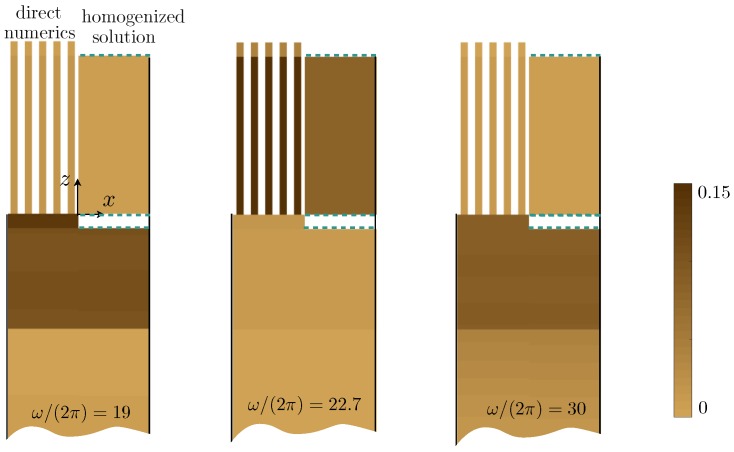
Surface densities of energy in the actual problem (computed numerically) and given by the homogenized solution at ω/(2π)=19 (corresponding to the resonance of the Love type with R=−1, the energy is stored in the layer and in the bottom layer), at ω/(2π)=22.7 (resonance with R=−1 of the SPP type; the energy is stored in the plate), and at ω/(2π), which is a standard case (R≃1, the energy is spread).

Eventually, we report in Figure 11 the surface density of energy *e* computed numerically ($e\;=\;\frac{1}{2}\;\left(\mu_{\mathrm{num},A}|\nabla u_{\mathrm{num}}{|}^{2}+\rho_{\mathrm{num},A}\omega^{2}{\left|u_{\mathrm{num}}\right|}^{2}\right)$ for A = S, L, b, P, t) and that of the effective problem (which varies with *z* only from (26)); it is worth noting that the energies $E_{t,b}$ do not give rise to surface density since they are defined along lines. We recover the observations of Figure 10: at a resonance of the layer (ω/(2π)=19), almost all the energy is stored in the layers; at a resonance of the plates (ω/(2π)=19), it is stored in the plates; and otherwise, it is equally distributed.

## 4. Concluding Remarks

We studied the problem of wave propagation in a geometry that combined two resonators, a soft layer in a substrate and an array of plates. In particular, we focused on the ability of thin heterogeneities at the endings of the plates to impact on the response of the system significantly. This was done thanks to asymptotic homogenization accounting for the boundary effects to be captured at the endings of the plates (e.g. foliages and roots for a model of trees). Such an analysis provides a simple effective model in which the region of the plates are replaced by a homogeneous highly anisotropic region and the effects of the heterogeneities were encapsulated in effective dynamic conditions. It was shown that the resulting effective model accurately predicts the dispersion relation of surface waves far beyond the quasi-static limit. These anti-plane shear waves share common features with Love waves in geophysics and surface plasmon polaritons (SPPs) in photonics; the dispersion relation of such hybridized Love waves was obtained in a closed form that allowed us to discriminate the role of the layer and that of the plates. Besides, we showed that the presence of heterogeneities at the decorative endings of the plates may affect significantly the characteristics of the surface waves. We also showed that the variations of the actual elastic energies (in the different regions) are accurately reproduced by the effective energies identified in the actual problem. In particular, the contributions of the effective interface and of the effective surface correspond to the actual energies stored in the thin regions containing the heterogeneities, and they disappear in the effective problem.

Our approach is useful for at least two reasons. On the one hand, it provides a simpler problem for which explicit solutions are available. This was illustrated in the present study where the model was shown to be very accurate up to frequencies corresponding to a ratio of the wavelength to the spacing close to one (typically in the layer and in the plates), where the long wavelength homogenization failed. Next, numerical resolution in the time domain may become intractable due to the separation of the scales associated with the typical wavelength, the array spacing, and the possible thinner scales in the heterogeneities. Eventually, the interest in dealing with effective problems was exemplified for scalar waves in a 2D geometry; it would be all the more evident for polarized elastic waves in 3D geometries. We finally note that our approach is well adapted to handle substrates with a gradient in elastic properties such as granular media [33,34].

## Figures and Tables

**Table 1:** Elastic material properties (in arbitrary units).

Substrate	Layer	Plate	Bottom	Top
$\mu_{S}$ = 2000	$\mu_{L}$ = 72	$\mu_{P}$ = 14.4	$\mu_{b}$ = 1600	$\mu_{t}$ = 1600
$\rho_{S}$ = 2000	$\rho_{L}$ = 1800	$\rho_{P}$ = 250	$\rho_{b}$ = 2500	$\rho_{t}$ = 2500
$c_{S}$ = 1000	$c_{L}$ = 200	$c_{P}$ = 240	$c_{b}$ = 800	$c_{t}$ = 800

**Table 2:** Homogenized coefficients entering in the effective conditions (3) in (5).

Coeff. in (5)	$L_{t}$	${\hat{\rho }}_{b}$		$\ell_{b}$	$L_{b}$	$l_{b}$
$h_{t}=0$	0	2500	$h_{b}=0$	0.1324	0.0120	0
$h_{t}=1$	10	2500	$h_{b}=1$	0.0511	22.2357	0

**Table 3:** Geometries of the four configurations, whose dispersion relations are given in Figure 7.

	$h_{L}$	$h_{b}$	$H_{L}$	$h_{P}$	$h_{t}$	$H_{P}$
Love	8	0	8	0	0	0
Modified Love	7	1	8	0	0	0
Case 1	8	0	8	12	0	12
Case 2	8	0	8	11	1	12
Case 3	7	1	8	12	0	12
Case 4	7	1	8	11	1	12

**Figure A1:**
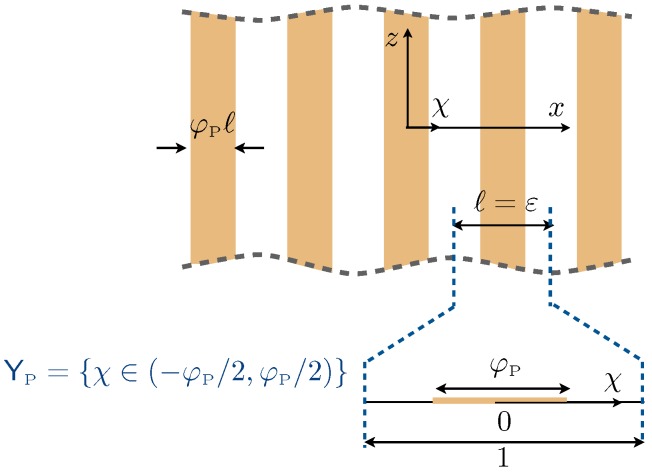
Homogenization in the bulk of the array. In the x=(x,z) coordinate, the array has a spacing ℓ=ε. The rescaling in the horizontal $\chi =x/\varepsilon \in Y_{P}$ coordinate is shown in the inset; $Y_{P}$ is a one-dimensional domain.

**Figure A2:**
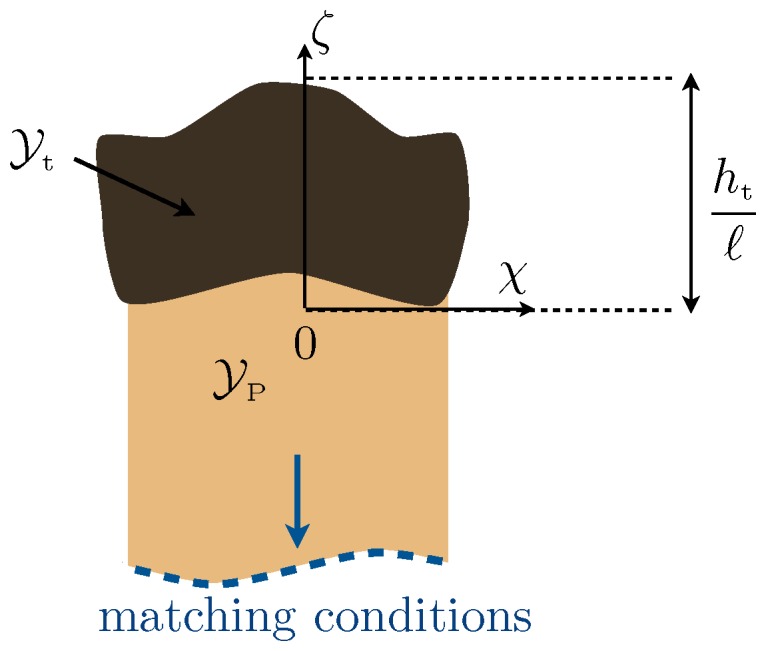
Elementary cell at the top of the plate in the χ=(χ,ζ) coordinate, in the unbounded two-dimensional region $Y=Y_{P}+Y_{t}$; the region of the plate is $Y_{P}=Y_{P}\times \left(-\infty ,0\right)$, and the heterogeneity $Y_{t}$ is bounded in $Y\times (0,h_{t})$, with $|Y_{t}|=\varphi_{t}h_{t}/\ell$.

## Appendix A. Asymptotic Analysis

In the asymptotic analysis that we shall conduct, we use the macroscopic (usual) coordinate x=(x,z), and we introduce the so-called microscopic coordinate χ=(χ,ζ), defined by:

(A1) $$\chi =\frac{x}{\ell },$$

where ℓ=ε is a small positive parameter (and small means small compared to the typical, or maximal, wavenumber imposed by the source of order unity). The analysis is firstly conducted in the region of the plates far from their endings. Afterwards specific analyses are conducted in the vicinity of plate endings to account for the boundary layer effects.

### Appendix A.1. The Homogenized Wave Equation

In the region of the plates, the wavefields vary over long distances in the two directions *x* and *z*; these long distance variations are accounted for by x. Next, short distance variations of the fields occur within a single plate, which are accounted for thanks to the additional coordinate χ; note that ζ is not needed since the plates are invariant along *z* (Figure A1).

Thus, the differential operator reads as:

$$\nabla \to \frac{e_{x}}{\varepsilon }\frac{\partial }{\partial \chi }+\nabla_{x},$$

which will be used in the plates where:

(A2) $$\sigma =\mu_{P}\nabla u,\;\rho_{P}\frac{\partial^{2}u}{\partial t^{2}}=\mathrm{div}\sigma ,$$

apply, owing to the expansions:

(A3) $$u=u^{0}\left(x,\chi ,t\right)+\varepsilon u^{1}\left(x,\chi ,t\right)+\cdots ,\;\sigma =\sigma^{0}\left(x,\chi ,t\right)+\varepsilon \sigma^{1}\left(x,\chi ,t\right)+\cdots ,$$

with $\chi \in Y_{P}$. During the homogenization procedure, the coordinate χ aims to disappear, and we shall see that the homogenized wave equation in (2) involves the effective fields $\langle u^{n}\rangle$ and $\langle \sigma^{n}\rangle$ defined by:

(A4) $$\langle u^{n}\rangle \left(x,t\right)\equiv \frac{1}{\varphi_{P}}\;\int_{Y_{P}}u^{n}\left(x,\chi ,t\right)d\chi ,\;\langle \sigma^{n}\rangle \left(x,t\right)\equiv \int_{Y_{P}}\sigma^{n}\left(x,\chi ,t\right)d\chi .$$

Doing so, we anticipate the macroscopic equilibrium of the forces by implicitly extending the stress by zero in $Y\backslash Y_{P}$, with Y={χ∈(−1/2,1/2)}.

We start the analysis at the leading order in 1/ε, with $\frac{\partial u^{0}}{\partial \chi }=\frac{\partial \sigma_{x}^{0}}{\partial \chi }=0$; hence $u^{0}\left(x,t\right)$ and $\sigma_{x}^{0}\left(x,t\right)$ do not depend on χ. It follows that $\sigma_{x}^{0}$ is constant in $Y_{P}$, and as it vanishes at the boundaries with air at $\chi =\pm \varphi_{P}/2$, it is zero everywhere in $Y_{P}$. Thus, we have:

(A5) $$\sigma_{x}^{0}\left(x,t\right)=0,\;u^{0}\left(x,t\right).$$

At the order $\varepsilon^{0}$ and accounting for (A5), we have:

(A6) $$\sigma_{z}^{0}\left(x,\chi ,t\right)=\mu_{P}\frac{\partial u^{0}}{\partial z}\left(x,t\right),\;\rho_{P}\frac{\partial^{2}u^{0}}{\partial t^{2}}\left(x,t\right)=\frac{\partial \sigma_{z}^{0}}{\partial z}\left(x,t\right)+\frac{\partial \sigma_{x}^{1}}{\partial \chi }\left(x,\chi ,t\right),\;\mathrm{in}\;Y_{P}.$$

It follows that $\sigma_{z}^{0}\left(x,t\right)$ does not depend on χ and $\sigma_{x}^{1}\left(x,t\right)=0$ (as for $\sigma_{x}^{0}$), and thus:

(A7) $$\langle \sigma_{z}^{0}\rangle \left(x,t\right)=\mu_{P}\varphi_{P}\frac{\partial u^{0}}{\partial z}\left(x,t\right),\;\rho_{P}\varphi_{P}\frac{\partial^{2}u^{0}}{\partial t^{2}}\left(x,t\right)=\frac{\partial \langle \sigma_{z}^{0}\rangle }{\partial z}\left(x,t\right),$$

by simply using that:

(A8) $$\langle \sigma_{z}^{0}\rangle \left(x,t\right)=\varphi_{P}\sigma_{z}^{0}\left(x,t\right),\;\sigma_{x}^{1}=0.$$

We now move on to the second order. Starting with (A5), hence $0=\sigma_{x}^{0}=\mu_{P}\left[\partial_{x}u^{0}\left(x,t\right)+\partial_{\chi }u^{1}\left(x,\chi ,t\right)\right]$, we deduce the displacement $u^{1}$ of the form:

(A9) $$u^{1}\left(x,\chi ,t\right)=-\chi \frac{\partial u^{0}}{\partial x}\left(x,t\right)+\langle u^{1}\rangle \left(x,t\right),\;\mathrm{in}\;Y_{P},$$

with the origin of χ such that 〈χ〉=0. It follows that:

(A10) $$\sigma_{z}^{1}\left(x,\chi ,t\right)=\mu_{P}\frac{\partial u^{1}}{\partial z}\left(x,\chi ,t\right)=\mu_{P}\left[-\chi \;\frac{\partial^{2}u^{0}}{\partial z\partial x}\left(x,t\right)+\frac{\partial \langle u^{1}\rangle }{\partial z}\left(x,t\right)\right],$$

which, after integration over $Y_{P}$ and thanks to 〈χ〉=0, leaves us with:

(A11) $$\langle \sigma_{z}^{1}\rangle \left(x,t\right)=\mu_{P}\varphi_{P}\frac{\partial \langle u^{1}\rangle }{\partial z}\left(x,t\right).$$

Eventually, the equation of equilibrium at order one reads as:

(A12) $$\rho_{P}\frac{\partial^{2}u^{1}}{\partial t^{2}}=div_{x}\sigma^{1}+\frac{\partial \sigma_{x}^{2}}{\partial \chi },\;\mathrm{in}\;Y_{P},$$

which after integration over $Y_{P}$ gives:

(A13) $$\rho_{P}\varphi_{P}\frac{\partial^{2}\langle u^{1}\rangle }{\partial t^{2}}=\frac{\partial \langle \sigma_{z}^{1}\rangle }{\partial z}\left(x,t\right).$$

The effective wave equations at the leading order (A7) Â and at order one (A11)–(A13) have the same forms; hence, up to $O(\varepsilon^{2})$, (u,σ) satisfies the effective equation announced in (2).

### Appendix A.2. The Boundary Condition at the Top of the Plates

The homogenized wave equation derived in the previous section has to be supplied with a boundary condition at the top of the plates. To derive this condition, we have to analyze the near (or evanescent) field excited in the vicinity of $z=h_{P}$ (Figure A2). In this region of small extent along *z*, the long distance variations of the macroscopic fields occur along *x* only (across the plates). Next, to describe the short distance variations of the evanescent field, we use the microscopic coordinates χ and:

(A14) $$\zeta =\frac{z-h_{P}}{\varepsilon }.$$

Accordingly, we consider the following asymptotic expansions:

(A15) $$u=v^{0}\left(x,\chi ,t\right)+\varepsilon v^{1}\left(x,\chi ,t\right)+\cdots ,\;\sigma =\tau^{0}\left(x,\chi ,t\right)+\varepsilon \tau^{1}\left(x,\chi ,t\right)+\cdots ,$$

with $\chi \in Y=Y_{t}\cup Y_{P}$ where $Y_{t}$ is the bounded region containing the heterogeneity (of vertical extent $h_{t}/\ell$ and $|Y_{t}|=\varphi_{t}h_{t}/\ell$), $Y_{P}=Y_{P}\times \left(-\infty ,0\right)$ is the unbounded region of the plate since in rescaled coordinate χ, and the bottom of the plate has been sent to −∞.

In Y, the fields (u,σ) satisfy:

(A16) $$\sigma =\mu \left(\chi \right)\nabla u,\;\rho \left(\chi \right)\frac{\partial^{2}u}{\partial t^{2}}=\mathrm{div}\sigma ,$$

with (μ(χ),ρ(χ)) varying within $Y_{t}$ depending on the characteristics of the heterogeneity and being equal to $\left(\mu_{P},\rho_{P}\right)$ in $Y_{P}$. The above system is complemented by a condition of zero normal stress at the boundaries in contact with air and conditions of continuity of displacement and normal stress at the interfaces between two elastic media. Eventually, boundary conditions are missing when ζ→−∞; these boundary conditions are obtained by imposing that the fields in (A15) match those defined in (A3), which hold far from the top of the plate. This is written in an intermediate region where $z\to h_{P}$ and ζ→−∞. Using that $z=h_{P}+\varepsilon \zeta$ in (A3) and re-expanding in Taylor expansions for small ε, we get the so-called matching conditions at each order. At the first and second orders and accounting for the fact that $u^{0}\left(x,t\right)$ and $\sigma^{0}\left(x,t\right)$ do not depend on χ from (A5) and (A8), we get:

(A17) $$\left\{\begin{array}{cc} u^{0}\left(x,h_{P},t\right)=\lim_{\zeta \to -\infty }v^{0}\left(x,\chi ,t\right), & \;\sigma^{0}\left(x,h_{P},t\right)=\lim_{\zeta \to -\infty }\tau^{0}\left(x,\chi ,t\right),\\ u^{1}\left(x,h_{P},\chi ,t\right)=\lim_{\zeta \to -\infty }\left(v^{1}\left(x,\chi ,t\right)-\zeta \frac{\partial u^{0}}{\partial z}\left(x,h_{P},t\right)\right), & \;\\ \sigma^{1}\left(x,h_{P},\chi ,t\right)=\lim_{\zeta \to -\infty }\left(\tau^{1}\left(x,\chi ,t\right)-\zeta \frac{\partial \sigma^{0}}{\partial z}\left(x,h_{P},t\right)\right). \end{array}\right.$$

We can now start the analysis, using $\nabla \to e_{x}\frac{\partial }{\partial x}+\frac{1}{\varepsilon }\nabla_{\chi }$ in (A16) along with (A15). At the leading order, we have $div_{\chi }\tau^{0}=0$, which after integration over Y and accounting for the boundary conditions and for the matching condition on $\sigma^{0}$ leaves us with:

(A18) $$0=\lim_{\zeta \to -\infty }\int_{Y_{P}}\tau_{z}^{0}\left(x,\chi ,t\right)d\chi =\sigma_{z}^{0}\left(x,h_{P},t\right).$$

At the leading order, the effective boundary condition is the usual stress-free condition, regardless of the presence of the heterogeneity. Hence, we move to the next order to get the boundary condition on $\sigma_{z}^{1}$, and to do so, we have to determine $\tau^{0}$. We start with $\nabla_{\chi }v^{0}=0$; hence, $v^{0}\left(x,t\right)$ is independent of χ; from the matching condition (A17) on $u^{0}$, we get that:

(A19) $$v^{0}\left(x,t\right)=u^{0}\left(x,h_{P},t\right).$$

This allows us to define the problem satisfied by $\left(v^{1},\tau^{0}\right)$ in χ coordinate, which reads as:

(A20) $$\left\{\begin{array}{c} div_{\chi }\tau^{0}=0,\;\tau^{0}\left(x,\chi ,t\right)=\mu \left(\chi \right)\left[\frac{\partial u^{0}}{\partial x}\left(x,h_{P},t\right)e_{x}+\nabla_{\chi }v^{1}\left(x,\chi ,t\right)\right],\;\;\mathrm{in}\;Y,\\ v^{1},\tau^{0}\cdot n,\;\mathrm{continuous}\;\mathrm{at}\;\mathrm{the}\;\mathrm{interfaces}\;\mathrm{between}\;\mathrm{two}\;\mathrm{elastic}\;\mathrm{media},\\ \tau^{0}\cdot n=0,\;\mathrm{at}\;\mathrm{the}\;\mathrm{boundaries}\;\mathrm{in}\;\mathrm{contact}\;\mathrm{with}\;\mathrm{air},\\ \lim_{\zeta \to -\infty }\tau^{0}=0. \end{array}\right.$$

For the limit ζ→−∞, we used in the matching condition (A17) for $\tau^{0}$ that $\sigma^{0}\left(x,h_{P},t\right)=0$ from (A5) and (A18). It is easy to check that the system (A20) has an explicit solution, which reads as:

(A21) $$\tau^{0}=0,\;v^{1}\left(x,\chi ,t\right)=-\chi \frac{\partial u^{0}}{\partial x}\left(x,h_{P},t\right)+{\hat{v}}^{1}\left(x,t\right),$$

where ${\hat{v}}^{1}\left(x,t\right)$ does not need to be specified, but it appears since $v^{1}$ is defined in (A20) up to a function of (x,t). Owing to the above results, the equation of equilibrium at the order $\varepsilon^{0}$ in (A16), specifically $\rho \frac{\partial^{2}v^{0}}{\partial t^{2}}=div_{\chi }\tau^{1}+\frac{\partial \tau_{x}^{0}}{\partial x}=0$, simplifies because of (A19) and (A21). After integration over Y and using that $\tau^{1}\cdot n$ is either zero (on the boundaries with the air) or continuous, we get:

(A22) $$\frac{\partial^{2}u^{0}}{\partial t^{2}}\left(x,h_{P},t\right)\int_{Y}\rho \left(\chi \right)d\chi =\int_{Y}div_{\chi }\tau^{1}d\chi =-\lim_{\zeta \to -\infty }\int_{Y_{P}}\tau_{z}^{1}\left(x,\chi ,t\right)d\chi .$$

Integrating over $Y_{P}$ the matching condition for $\sigma_{z}^{1}$ in (A17), along with $\partial_{z}\langle \sigma_{z}^{0}\rangle =\rho_{P}\varphi_{P}\partial_{tt}u^{0}$ from (A7), we find that (A22) can be written as:

(A23) $$\langle \sigma_{z}^{1}\rangle \left(x,h_{P},t\right)=-\frac{\partial \langle \sigma_{z}^{0}\rangle }{\partial z}\left(x,h_{P},t\right)\;\lim_{\zeta \to -\infty }\left(\zeta +\int_{Y}\frac{\rho (\chi )}{\rho_{P}\varphi_{P}}d\chi \right).$$

The integral of the mass density over Y is $\int_{Y}\rho \left(\chi \right)d\chi =\int_{Y_{t}}\rho \left(\chi \right)d\chi -\lim_{\zeta \to -\infty }\zeta \rho_{P}\varphi_{P}$, from which:

(A24) $$\langle \sigma_{z}^{1}\rangle \left(x,h_{P},t\right)=-\int_{Y_{t}}\frac{\rho (\chi )}{\rho_{P}\varphi_{P}}d\chi \;\frac{\partial \langle \sigma_{z}^{0}\rangle }{\partial z}\left(x,h_{P},t\right).$$

The above expression is valid if one considers a heterogeneity with varying mass density, and in the case of uniform mass density $\rho \left(\chi \right)=\rho_{t}$, it simplifies to:

(A25) $$\langle \sigma_{z}^{1}\rangle \left(x,h_{P},t\right)=-\frac{h_{t}}{\ell }\frac{\rho_{t}\varphi_{t}}{\rho_{P}\varphi_{P}}\frac{\partial \langle \sigma_{z}^{0}\rangle }{\partial z}\left(x,h_{P},t\right).$$

Eventually, making use of $\sigma_{z}^{0}\left(x,h_{P},t\right)=0$ in (A18) and of (A25), we get that $\left(\sigma_{z}^{0}+\varepsilon \langle \sigma_{z}^{1}\rangle \right)$ (with ε=ℓ); hence $\sigma_{z}$ up to $O(\varepsilon^{2})$ satisfies the boundary condition announced in (3).

### Appendix A.3. Jump Conditions

The analysis of the problem in the vicinity of the heterogeneity at the bottom of the plates (Figure A3) is similar to that conducted in the previous section. The elementary cell in which the analysis is conducted is unbounded for ζ→±∞ since in the microscopic coordinate, the top of the plate and the lower interface of the layer were set to infinity. We use the same expansions as in (A15), and for simplicity, we keep the same notations, with χ=(χ,ζ) and:

(A26) $$\chi =\frac{x}{\varepsilon },\;\zeta =\frac{z}{\varepsilon }.$$

In the present case, the elementary cell involves a part of ζ<0 where we impose that $\left(v^{n},\tau^{n}\right)$ in (A15) are periodic with respect to χ∈Y.

As in the previous section, the matching conditions tell us that the solution in Y when ζ→±∞ matches the solution valid far from the bottom of the plates $z\in (-h_{b},0)$. When ζ→+∞, it matches (A3), which holds in the plates; when ζ→−∞, it matches the solution in the layer. As the layer is a homogeneous region, the expansion of the solution (u,σ) is trivial: of the same form as in (A3) with all the terms $\left(u^{n},\sigma^{n}\right)$ being a function of (x,t) only. We thus get that:

(A27) $$\left\{\begin{array}{cc} u^{0}\left(x,0^{\pm },t\right)=\lim_{\zeta \to \pm \infty }v^{0}\left(x,\chi ,t\right), & \;\sigma^{0}\left(x,0^{\pm },t\right)=\lim_{\zeta \to \pm \infty }\tau^{0}\left(x,\chi ,t\right),\\ u^{1}\left(x,0^{-},t\right)=\lim_{\zeta \to -\infty }\left(v^{1}\left(x,\chi ,t\right)-\zeta \frac{\partial u^{0}}{\partial z}\left(x,0^{-},t\right)\right),\\ u^{1}\left(x,0^{+},\chi ,t\right)=\lim_{\zeta \to +\infty }\left(v^{1}\left(x,\chi ,t\right)-\zeta \frac{\partial u^{0}}{\partial z}\left(x,0^{+},t\right)\right),\\ \;\sigma^{1}\left(x,0^{-},t\right)=\lim_{\zeta \to -\infty }\left(\tau^{1}\left(x,\chi ,t\right)-\zeta \frac{\partial \sigma^{0}}{\partial z}\left(x,0^{-},t\right)\right),\\ \;\sigma^{1}\left(x,0^{+},\chi ,t\right)=\lim_{\zeta \to +\infty }\left(\tau^{1}\left(x,\chi ,t\right)-\zeta \frac{\partial \sigma^{0}}{\partial z}\left(x,0^{+},t\right)\right). \end{array}\right.$$

For the limit of $\left(v^{n},\tau^{n}\right)$ (n=0,1) when ζ→−∞, we used that all the $\left(u^{n},\sigma^{n}\right)$ do not depend on χ in the layer. For the limit of $\left(v^{n},\tau^{n}\right)$ (n=0,1) when ζ→+∞, we used that $\left(u^{0},\sigma^{0}\right)$ do not depend on χ in the plates from (A5) and (A8), but $\left(u^{1},\sigma^{1}\right)$ do.

We can now start the analysis, using as in the previous section, the differential operator $\nabla \to e_{x}\frac{\partial }{\partial x}+\frac{1}{\varepsilon }\nabla_{\chi }$ and the expansions (A15) in:

(A28) $$\sigma =\mu \left(\chi \right)\nabla u,\;\rho \left(\chi \right)\frac{\partial^{2}u}{\partial t^{2}}=\mathrm{div}\sigma ,$$

with (μ(χ),ρ(χ)) varying within $Y_{b}=\left\{\chi \in Y\times \left(-\infty ,0\right)\right\}$ and being equal to $\left(\mu_{P},\rho_{P}\right)$ in $Y_{P}=\left\{\chi \in Y_{P}\times \left(0,+\infty \right)\right\}$. As previously, the leading order starts with $\nabla_{\chi }v^{0}=0$; hence: $v^{0}$ is independent of χ, and from (A27), $v^{0}\left(x,t\right)=u^{0}\left(x,0^{\pm },t\right)$. Next, integrating the relation $div_{\chi }\tau^{0}=0$ over Y along with the (A27) leaves us with:

(A29) $$v^{0}\left(x,t\right)=u^{0}\left(x,0,t\right),\;{⟦\langle \sigma_{z}^{0}\rangle ⟧}_{0}={⟦u^{0}⟧}_{0}=0,$$

where we have defined ${⟦w⟧}_{0}=w\left(x,0^{+},t\right)-w\left(x,0^{-},t\right)$ at this stage. At the leading order, the continuity of the displacement and of the normal stress apply regardless of the presence of the heterogeneities. Thus, we move to the next order, and as in the previous section, we need to define the problem on $\left(v^{1},\tau^{0}\right)$, which reads as:

(A30) $$\left\{\begin{array}{c} div_{\chi }\tau^{0}=0,\;\tau^{0}=\mu \left(\chi \right)\left(\nabla_{\chi }v^{1}+\frac{\partial u^{0}}{\partial x}\left(x,0,t\right)\;e_{x}\right),\;\;\mathrm{in}\;Y,\\ v^{1},\tau^{0}\cdot n,\;\mathrm{continuous}\;\mathrm{at}\;\mathrm{the}\;\mathrm{interfaces}\;\mathrm{between}\;\mathrm{two}\;\mathrm{elastic}\;\mathrm{media},\\ \tau^{0}\cdot n=0,\;at\chi =\pm \varphi_{P}/2,\;\mathrm{in}\;Y_{P},\\ v^{1},\tau^{0},\;\mathrm{one}\;\mathrm{periodic}\;\mathrm{with}\;\mathrm{respect}\;\mathrm{to}\;\chi \;\mathrm{in}\;Y_{b},\\ \lim_{\zeta \to -\infty }\nabla_{\chi }v^{1}=\frac{\langle \sigma_{z}^{0}\rangle \left(x,0,t\right)}{\mu_{L}}\;e_{z},\;\lim_{\zeta \to +\infty }\nabla_{\chi }v^{1}=\frac{\langle \sigma_{z}^{0}\rangle \left(x,0,t\right)}{\mu_{P}\varphi_{P}}\;e_{z}-\frac{\partial u^{0}}{\partial x}\left(x,0,t\right)\;e_{x}. \end{array}\right.$$

To find the above limits for ζ→±∞, we used the matching conditions for $\sigma^{0}$ in (A27) along with $\sigma^{0}\left(x,0^{-},t\right)=\langle \sigma_{z}^{0}\rangle \left(x,0,t\right)e_{z}+\mu_{L}\partial_{x}u^{0}\left(x,0,t\right)e_{x}$ (since in the layer, $\sigma_{z}^{0}=\langle \sigma_{z}^{0}\rangle$ and $\sigma_{x}^{0}=\mu_{L}\partial_{x}u^{0}$) and along with $\sigma^{0}\left(x,0^{+},t\right)=\langle \sigma_{z}^{0}\rangle \left(x,0,t\right)e_{z}/\varphi_{P}$ from (A5) and (A8).

The system (A30) is the counterpart of the system (A20) that we obtained at the top of the plate, but now, the solution is not trivial; hence, (A20) has to be solved numerically. However, instead of solving (A30) for a given scattering problem, that is for given external loadings $\left(\partial_{x}u^{0}\left(x,0,t\right),\langle \sigma_{z}^{0}\rangle \left(x,0,t\right)\right)$, we shall use that (A30) is linear with respect to those loadings. Specifically, we set:

(A31) $$\left\{\begin{array}{c} v^{1}=\frac{1}{\mu_{L}}\;\langle \sigma_{z}^{0}\rangle \left(x,0,t\right)V_{1}\left(\chi \right)+\frac{\partial u^{0}}{\partial x}\left(x,0,t\right)V_{2}\left(\chi \right)+{\hat{v}}^{1}\left(x,t\right),\\ \tau^{0}=\frac{\mu (\chi )}{\mu_{L}}\;\langle \sigma_{z}^{0}\rangle \left(x,0,t\right)\nabla_{\chi }V_{1}\left(\chi \right)+\mu \left(\chi \right)\frac{\partial u^{0}}{\partial x}\left(x,0,t\right)\nabla_{\chi }\left(V_{2}\left(\chi \right)+\chi \right), \end{array}\right.$$

and it is sufficient that $\left(V_{1},V_{2}\right)$ satisfy (4) to ensure that $v^{1}$ satisfies (A30). The advantage is obvious: as $\left(V_{1},V_{2}\right)$ satisfy static problems, they can be computed once and for all, independently of the scattering problem that will be considered afterwards. We shall see that these elementary solutions provide the coefficients defined in (5).

From (4), $V_{1}$ has a linear behavior in ζ for ζ→±∞, and $V_{2}$ is linear in χ when ζ→+∞. As $V_{1},V_{2}$ in (4) are defined up to a constant, we set the constant at zero for ζ→−∞ and define:

(A32) $$\left\{\begin{array}{cc} \lim_{\zeta \to -\infty }\left(V_{1}-\zeta \right)=0, & \;\lim_{\zeta \to -\infty }V_{2}=0,\\ \lim_{\zeta \to +\infty }\left(V_{1}-\frac{\mu_{L}}{\varphi_{P}\mu_{P}}\zeta \right)=\alpha_{1}, & \;\lim_{\zeta \to +\infty }\left(V_{2}+\chi \right)=\alpha_{2}. \end{array}\right.$$

The jump of $\langle u^{1}\rangle$ is obtained using the matching conditions (A27) for $u^{1}$, with $v^{1}$ in (A31) along with (A32). Using in addition that $\langle \sigma_{z}^{0}\rangle =\mu_{P}\varphi_{P}\partial_{z}$, which holds at $z=0^{+}$ from (A7), and $\langle \sigma_{z}^{0}\rangle =\sigma_{z}^{0}=\mu_{L}\partial_{z}u^{0}$, which holds at $z=0^{-}$, we easily get that:

(A33) $$u^{1}\left(x,0^{-},\chi ,t\right)={\hat{v}}^{1}\left(x,t\right),\;\mathrm{and}\;u^{1}\left(x,0^{+},\chi ,t\right)=\frac{\alpha_{1}}{\mu_{L}}\;\langle \sigma_{z}^{0}\rangle \left(x,0,t\right)+\left(\alpha_{2}-\chi \right)\frac{\partial u^{0}}{\partial x}\left(x,0,t\right)+{\hat{v}}^{1}\left(x,t\right)$$

(incidentally, we recover that $u^{1}$ is linear w.r.t. χ as in (A9)). After integration over $Y_{P}$ (with 〈χ〉=0), we get:

(A34) $${⟦\langle u^{1}\rangle ⟧}_{0}=\frac{\alpha_{1}}{\mu_{L}}\;\langle \sigma_{z}^{0}\rangle \left(x,0,t\right)+\alpha_{2}\frac{\partial u^{0}}{\partial x}\left(x,0,t\right).$$

To get the jump on $\langle \sigma_{z}^{1}\rangle$, we use the equation of equilibrium in (A16) at order zero integrated over Y, specifically:

(A35) $$\frac{\partial^{2}u^{0}}{\partial t^{2}}\left(x,0,t\right)\int_{Y}\rho \left(\chi \right)\;d\chi =\int_{Y}\left(div_{\chi }\tau^{1}+\frac{\partial \tau_{x}^{0}}{\partial x}\right)\;d\chi$$

where we use that $v^{0}=u^{0}\left(x,0,t\right)$ from (A29). We use (A27) along with (A31), and we account for the fact that $\int_{Y_{b}}\partial_{\chi }V_{2}\;d\chi$ and $\int_{Y_{P}}\partial_{\chi }\left(V_{2}+\chi \right)d\chi$ are bounded and that $\int_{Y_{P}}d\chi =\varphi_{P}\lim_{\zeta \to +\infty }\zeta$ and $\int_{Y_{b}}d\chi =\lim_{\zeta \to +\infty }\zeta$. We also use that $\rho_{P}\partial_{tt}u^{0}=\partial_{z}\sigma_{z}^{0}$ in the plate (from (A7) and (A8), which holds for $z=0^{+}$) and that $\rho_{L}\partial_{tt}u^{0}-\mu_{L}\partial_{xx}u^{0}-\partial_{z}\sigma_{z}^{0}=0$ in the layer (from (1), which holds for $z=0^{-}$). Eventually, for constant $\rho =\rho_{b}$ and $\mu =\mu_{b}$ in the heterogeneity, we use that $\int_{Y_{b}}\left(\rho -\rho_{L}\right)d\chi =\varphi_{b}h_{b}/\ell \left(\rho_{b}-\rho_{L}\right)$, $\int_{Y_{b}}\left(\mu -\mu_{L}\right)d\chi =\varphi_{b}h_{b}/\ell \left(\mu_{b}-\mu_{L}\right)$. This leaves us with the jump in $\sigma_{z}^{1}$ of the form:

(A36) $${⟦\langle \sigma_{z}^{1}\rangle ⟧}_{0}=-\beta_{1}\frac{\partial \langle \sigma_{z}^{0}\rangle }{\partial x}\left(x,0,t\right)-\mu_{L}\beta_{2}\frac{\partial^{2}u^{0}}{\partial x^{2}}\left(x,0,t\right)+\frac{\varphi_{b}h_{b}}{\ell }\left(\rho_{b}-\rho_{L}\right)\frac{\partial^{2}u^{0}}{\partial t^{2}}\left(x,0,t\right),$$

with:

(A37) $$\beta_{1}=\int_{Y}\frac{\mu }{\mu_{L}}\frac{\partial V_{1}}{\partial \chi }d\chi ,\;\beta_{2}=\int_{Y_{P}}\frac{\mu }{\mu_{L}}\frac{\partial (V_{2}+\chi )}{\partial \chi }d\chi +\int_{Y_{b}}\frac{\mu }{\mu_{L}}\frac{\partial V_{2}}{\partial \chi }d\chi +\left(\frac{\mu_{b}}{\mu_{L}}-1\right)\frac{\varphi_{b}h_{b}}{\ell }.$$

We shall express the jump conditions obtained in (A29) and in (A34)–(A36) in a different form, but equivalent up to $O(\varepsilon^{2})$. Specifically, we want the jump between $-h_{b}$ and zero (we shall comment on this choice later on). With ℓ=ε and $h_{b}=O\left(\ell \right)$, we can use the Taylor expansion of $w^{0}\left(x,0^{-},t\right)=w^{0}\left(x,-h_{b},t\right)+h_{b}\;\partial_{z}w^{0}\left(x,0^{-},t\right)+O\left(\varepsilon^{2}\right)$ for $w^{0}=u^{0},\langle \sigma_{z}^{0}\rangle$ to get the jump of $w=w^{0}+\varepsilon w^{1}$ defined as $⟦w⟧=w\left(x,0^{+},t\right)-w\left(x,-h_{b},t\right)$ (as in (3)). From (A29) along with (A34) Â and (A36), we get:

(A38) $$⟦u^{0}+\varepsilon \langle u^{1}\rangle ⟧=\frac{\alpha_{1}\ell +h_{b}}{\mu_{L}}\;\langle \sigma_{z}^{0}\rangle \left(x,0,t\right)+\alpha_{2}\ell \;\frac{\partial u^{0}}{\partial x}\left(x,0,t\right)+O\left(\ell^{2}\right),$$

where we used that $\langle \sigma_{z}^{0}\rangle =\sigma_{z}^{0}=\mu_{L}\partial_{z}u^{0}$. Replicating this for the jump in the normal stress, we get:

(A39) $$⟦\sigma_{z}^{0}+\varepsilon \langle \sigma_{z}^{1}\rangle ⟧=-\beta_{1}\ell \frac{\partial \sigma_{z}^{0}}{\partial x}\left(x,0,t\right)-\mu_{L}\left(h_{b}+\beta_{2}\ell \right)\frac{\partial^{2}u^{0}}{\partial x^{2}}\left(x,0,t\right)+h_{b}\left[\varphi_{b}\rho_{b}+\left(1-\varphi_{b}\right)\rho_{L}\right]\frac{\partial^{2}u^{0}}{\partial t^{2}}\left(x,0,t\right)+O\left(\ell^{2}\right),$$

where we used that $\partial_{z}\langle \sigma_{z}^{0}\rangle =\partial_{z}\sigma_{z}^{0}=-\mu_{L}\partial_{xx}u^{0}+\rho_{L}\partial_{tt}u^{0}$ (and it will be shown in Appendix B.1 that $\beta_{1}=-\alpha_{2}$). Eventually, we define $\bar{w}=\frac{1}{2}\left(w\left(x,-h_{b},t\right)+w\left(x,0^{+},t\right)\right)$; with $\ell w^{0}\left(x,0,t\right)=\ell \bar{w}+O\left(\ell^{2}\right)$ in the right hand side terms, the jumps in (A38) and (A39) are equivalent to that written in (3) omitting $O(\ell^{2})$, and with:

(A40) $$\ell_{b}=\alpha_{1}\ell +h_{b},\;l_{b}=\alpha_{2}\ell ,\;L_{b}=h_{b}+\beta_{2}\ell ,$$

according to (5) along with (A32) and (A37). We made a choice on the expression of the jump conditions, and we shall see that this guaranties that the energy in the effective problem is a definite positive quadratic form. Note that we also made a choice on the position of the effective boundary condition on the top of the plate; it is possible to choose a different position in the vicinity of the order of *ℓ* of that one; however, as already stressed, if all the resulting effective problems are equivalent up to $O(\ell^{2})$, all of them do not guaranty proper energy of the effective problem; see, e.g., [31,32].

## Appendix B. Properties of the Effective Parameters

Here, we shall prove three properties that have been used in the previous Appendix: (1) In (A39), we used that $\alpha_{2}+\beta_{1}=0$. Furthermore, the effective energy $E_{\mathrm{eff}}$ in (10) is a definite positive quadratic form if: (2) $\ell_{b}>0$ and (3) $L_{b}>0$, with $\left(\ell_{b},L_{b}\right)$ defined in (A40).

### Appendix B.1. α*_2_* + β*_1_* = *0*

The parameters $\left(\alpha_{2},\beta_{1}\right)$ are defined by $\alpha_{2}=\lim_{\zeta \to +\infty }\left(V_{2}+\chi \right)$ (see (A32)) and $\beta_{1}=\int_{Y}\frac{\mu }{\mu_{L}}\frac{\partial V_{1}}{\partial \chi }d\chi$ (see (A37)), with $V_{1}$ and $V_{2}$ satisfying the elementary problems: (4) (note that $\ell_{b}=\ell \alpha_{2}$ in (5)). We start with:

(A41) $$0=\int_{Y}V_{2}\;\mathrm{div}\left(\frac{\mu }{\mu_{L}}\nabla V_{1}\right)d\chi =-\int_{Y}\frac{\mu }{\mu_{L}}\nabla V_{1}\cdot \nabla V_{2}\;d\chi +\alpha_{2},$$

where we used that all the boundary terms $\mu V_{2}\nabla V_{1}\cdot n$ on ∂Y vanish except at ζ→+∞ where $\int_{Y_{P}}\frac{\mu }{\mu_{L}}\;V_{2}\nabla V_{1}\cdot e_{z}\;d\chi =\int_{Y_{P}}\frac{1}{\varphi_{P}}\;\left(\alpha_{2}-\chi \right)\;d\chi =\alpha_{2}$ since $\mu =\mu_{P}$ and 〈χ〉=0. Indeed, $V_{2}$ and $\mu \nabla V_{1}\cdot n$ are continuous at the interface between two elastic media; next, $\mu \nabla V_{1}\cdot n=0$ at the boundaries in contact with air; eventually, for ζ→−∞, $V_{2}$ vanishes. Next, considering:

(A42) $$0=\int_{Y}V_{1}\;\mathrm{div}\left(\frac{\mu }{\mu_{L}}\nabla \left(V_{2}+\chi \right)\right)d\chi =-\int_{Y}\frac{\mu }{\mu_{L}}\nabla V_{1}\cdot \nabla V_{2}\;d\chi -\beta_{1},$$

and here, all the boundary terms vanish since $\mu \nabla (V_{2}+\chi )\cdot n$ is continuous or vanish and for ζ→±∞$\nabla \left(V_{2}+\chi \right)\cdot \left(\pm e_{z}\right)=0$. It follows that:

(A43) $$\alpha_{2}=-\beta_{1}=\int_{Y}d\chi \;\frac{\mu }{\mu_{L}}\nabla V_{1}\cdot \nabla V_{2}.$$

### Appendix B.2. ℓ_b_ > *0*

To show that $\ell_{b}>0$ or equivalently $\alpha_{1}+h_{b}/\ell \geq 0$ from (A40), we rely on the variational formulation of the elementary problem on square integrable field $V=V_{1}-H$ with H(χ,ζ<0)=ζ and $H\left(\chi ,\zeta >0\right)=\frac{\mu_{L}}{\varphi_{P}\mu_{P}}\zeta$. Thus, *V* satisfies $\mathrm{div}\left(\left(\mu /\mu_{L}\right)\nabla \left(V+H\right)\right)=0$ with $\lim_{\zeta \to \pm \infty }\nabla V=0$, from (4), and (V+H), $\left(\mu /\mu_{L}\right)\nabla \left(V+H\right)\cdot n$ are continuous at the interfaces between two elastic media and vanish at the boundaries in contact with air.

We now define the set V of admissible fields $\tilde{V}$ being continuous with $\nabla \tilde{V}\to 0$ for |ζ|→+∞. Next, we introduce the energy defined over V by:

(A44) $$\begin{array}{cc} \tilde{V}\in V, & E\left(\tilde{V}\right)=\int_{Y}\frac{\mu }{\mu_{L}}\left(\frac{1}{2}{\left|\nabla \tilde{V}\right|}^{2}+\nabla \tilde{V}\cdot \nabla H\right)d\chi -\alpha \left(\tilde{V}\right),\\ & \mathrm{with}\;\alpha \left(\tilde{V}\right)=\frac{1}{\varphi_{P}}\int_{Y_{P}}\;\tilde{V}\left(\chi ,+\infty \right)d\chi -\int_{Y}\tilde{V}\left(\chi ,-\infty \right)d\chi . \end{array}$$

One can show using standard arguments of calculus of variations that the minimizer *V* of *E* defined by:

(A45) $$V=\underset{\tilde{V}\in V}{arg min}E\left(\tilde{V}\right),$$

corresponds to $V=V_{2}-H$ with $\alpha \left(V\right)=\alpha_{2}$ since, by the definition of *V* and from (A32), $\lim_{\zeta \to -\infty }V=0$ and $\lim_{\zeta \to +\infty }V=\alpha_{1}$. To conclude, we need the expression of E(V). We multiply the relation $\mathrm{div}\left(\left(\mu /\mu_{L}\right)\nabla \left(V+H\right)\right)=0$ by *V*, then by *H*, and integrate by parts. We get that $\int_{Y}\frac{\mu }{\mu_{L}}{\left|\nabla V\right|}^{2}d\chi +\int_{Y}\frac{\mu }{\mu_{L}}\nabla V\cdot \nabla H\;d\chi =\alpha_{1}$ and, after straightforward calculations, that $\int_{Y}d\chi \frac{\mu }{\mu_{L}}\nabla V\cdot \nabla H=\varphi_{b}\frac{h_{b}}{\ell }\left(1-\frac{\mu_{b}}{\mu_{L}}\right)$. It follows that, from (A44), we have:

(A46) $$E\left(V\right)=\frac{1}{2}\left(\varphi_{b}\frac{h_{b}}{\ell }\left(1-\frac{\mu_{b}}{\mu_{L}}\right)-\alpha_{1}\right).$$

Thus, by bounding the energy E(V), we shall get a lower bound for $\alpha_{1}$, hence for $\ell_{b}=\alpha_{1}\ell +h_{b}$. To do so, we chose a test field $\tilde{V}$ being piecewise linear along ζ, with $\tilde{V}\left(\chi \right)=f\left(\zeta \right)$ and f(ζ)=0, $b\ell \left(\zeta +h_{b}/\ell \right)/h_{b}$, *b* for $\zeta \in (-\infty ,-h_{b})$, $\left(-h_{b},0\right)$, and (0,+∞), respectively. At this stage, *b* is a free parameter that we shall fix to minimize $E(\tilde{V})$. It is easy to see that:

(A47) $$E\left(\tilde{V}\right)=\frac{h_{b}}{\ell }\left[\frac{1}{2}\left(\varphi_{b}\frac{\mu_{b}}{\mu_{L}}+1-\varphi_{b}\right){\left(\frac{b\ell }{h_{b}}\right)}^{2}+\varphi_{b}\left(\frac{\mu_{b}}{\mu_{L}}-1\right)\left(\frac{b\ell }{h_{b}}\right)\right],$$

whose minimum with respect to *b* is obtained for $b^{*}=-\left(h_{b}/\ell \right)\varphi_{b}\left(\mu_{b}/\mu_{L}-1\right)/\left(\varphi_{b}\mu_{b}/\mu_{L}+1-\varphi_{b}\right)$ and provides:

(A48) $$\min E\left(\tilde{V}\right)=-\frac{h_{b}}{2\ell }\;\frac{\varphi_{b}^{2}{\left(\mu_{b}/\mu_{L}-1\right)}^{2}}{\varphi_{b}\mu_{b}/\mu_{L}+1-\varphi_{b}}$$

It is now sufficient to use (A45) along with (A46) and (A48) to find that:

(A49) $$\ell_{b}\geq \frac{h_{b}}{\varphi_{b}\frac{\mu_{b}}{\mu_{L}}+1-\varphi_{b}}>0.$$

### Appendix B.3. L_b_ ≥ *0*

To obtain a lower bound on $L_{b}$, we rely on the variational formulation of the elementary problem on $V_{2}$. First, we define the set T of admissible fields $\tilde{\tau }$ such that:

(A50) $$\left\{\begin{array}{c} \tilde{\tau }\cdot n,\;\mathrm{continuous}\;\mathrm{at}\;\mathrm{the}\;\mathrm{interfaces}\;\mathrm{between}\;\mathrm{two}\;\mathrm{elastic}\;\mathrm{media},\\ \tilde{\tau }\cdot n=0,\;at\chi =\pm \varphi_{P}/2,\;\mathrm{in}\;Y_{P},\\ \tilde{\tau },\;\mathrm{one}\;\mathrm{periodic}\;\mathrm{with}\;\mathrm{respect}\;\mathrm{to}\;\chi \;\mathrm{in}\;Y_{b},\\ \lim_{\zeta \to -\infty }\tilde{\tau }=e_{x},\;\lim_{\zeta \to +\infty }\tilde{\tau }=0. \end{array}\right.$$

Next, we introduce the complementary energy defined over T:

(A51) $$\tilde{\tau }\in T,\;E^{*}\left(\tilde{\tau }\right)=\int_{Y_{b}}\frac{\mu }{\mu_{L}}{\left|\tilde{\tau }-\frac{\mu }{\mu_{L}}e_{x}\right|}^{2}\;d\chi +\int_{Y_{P}}\left(\frac{\mu_{L}}{\mu }{\left|\tilde{\tau }\right|}^{2}-2\tilde{\tau }\cdot e_{x}\right)d\chi .$$

One can show from standard arguments of the calculus of variations that the minimizer τ of $E^{*}$ defined by:

(A52) $$\tau =\underset{\tilde{\tau }\in T}{arg min}E^{*}\left(\tilde{\tau }\right),$$

corresponds to the stress field associated with the solution $V_{2}$, that is to say:

(A53) $$\tau =\frac{\mu }{\mu_{L}}\left(\nabla_{\chi }V_{2}+e_{x}\right).$$

Now, multiplying by $V_{2}$ the equilibrium equation (4) associated with $V_{2}$, integrating by parts, and using (A51) and (A53), we find the relation:

(A54) $$\frac{L_{b}}{\ell }-\frac{h_{b}}{\ell }\left(\varphi_{b}\frac{\mu_{b}}{\mu_{L}}+\left(1-\varphi_{b}\right)\right)=\int_{Y_{P}}\frac{\mu }{\mu_{L}}\frac{\partial (V_{2}+\chi )}{\partial \chi }d\chi +\int_{Y_{b}}\frac{\mu }{\mu_{L}}\frac{\partial V_{2}}{\partial \chi }d\chi =-E^{*}\left(\tau \right).$$

Thus, by bounding the complementary energy of the solution $E^{*}\left(\tau \right)$, we shall get a bound on the left hand side in (A54), hence on $L_{b}$ given by (5). To do so, we chose the piecewise constant test field $\tilde{\tau }$ such that $\tau \left(\chi \right)=g\left(\zeta \right)e_{x}$ with g(ζ)=1, *a*, and 0 for $\zeta \in (-\infty ,-h_{b}/\ell )$, $\left(-h_{b}/\ell ,0\right)$, and (0,+∞), respectively. At this stage, *a* is a free parameter that we shall fix to minimize $E^{*}\left(\tilde{\tau }\right)$. Calculating the energy of such a test field gives:

(A55) $$E^{*}\left(\tilde{\tau }\right)={\left(a-\frac{\mu_{b}}{\mu_{L}}\right)}^{2}\frac{\mu_{L}}{\mu_{b}}\varphi_{b}h_{b}+{\left(a-1\right)}^{2}h_{b}\left(1-\varphi_{b}\right).$$

Now, minimizing the energy with respect to *a* gives the optimum $a^{*}={\left(\varphi_{b}\frac{\mu_{L}}{\mu_{b}}+1-\varphi_{b}\right)}^{-1}$. By injecting $a^{*}$ in (A55) and using (A54), we finally obtain:

(A56) $$L_{b}\geq \frac{h_{b}}{\varphi_{b}\frac{\mu_{L}}{\mu_{b}}+1-\varphi_{b}}>0.$$
